# Integrated mesenchymal and extracellular cues drive bioengineered liver tissue formation and function

**DOI:** 10.1016/j.mtbio.2026.103328

**Published:** 2026-06-09

**Authors:** Shicheng Ye, Zhenguo Wang, Maarten Dirk Delemarre, Nalan Liv, Antoinette van den Dikkenberg, Tina Vermonden, Luc van der Laan, Jos Malda, Bart Spee, Frank G. Van Steenbeek, Kerstin Schneeberger-Verjaal

**Affiliations:** a Department of Clinical Sciences, Faculty of Veterinary Medicine, Utrecht University, Uppsalalaan 8, Utrecht, CT, 3584, Netherlands; b Division of Pharmacology, Department of Pharmaceutical Sciences, Faculty of Sciences, Utrecht Institute for Pharmaceutical Sciences, Utrecht University, Utrecht, Netherlands; c Department of Experimental Research, State Key Laboratory of Oncology in South China, Guangdong Provincial Clinical Research Center for Cancer, Sun Yat-sen University Cancer Center, Guangzhou, 510060, China; d Center for Molecular Medicine, University Medical Center Utrecht, Utrecht University, Utrecht, Netherlands; e Division of Pharmaceutics, Department of Pharmaceutical Sciences, Faculty of Sciences, Utrecht Institute for Pharmaceutical Sciences, Utrecht University, Utrecht, Netherlands; f Department of Surgery Erasmus MC-University Medical Center, Dr. Molewaterplein 40, Rotterdam, GD, 3015, Netherlands; g Department of Orthopaedics, University Medical Center Utrecht, Utrecht University, Utrecht, CX, 3584, Netherlands

**Keywords:** Bioengineering, Hydrogel, Intrahepatic cholangiocyte organoid, Hepatic stellate cell, Co-culture, Dynamic suspension culture, In vitro models

## Abstract

Human tissue engineering holds great promise for creating physiological models while facing challenges replicating natural complexity, including cellular and extracellular cues. Current approaches often miss the incorporation of major bioengineering factors (*i.e.*, cellular complexity, well-defined extracellular matrix (ECM) mimicry, dynamic stimuli). We bioengineered liver tissues (BLTs) utilizing human intrahepatic cholangiocyte organoids (ICOs), hepatic stellate cells (HSCs), and mesenchymal stromal cells (MSCs), a synthetic polyisocynide (PIC)- based hydrogel, and dynamic suspension culture (DS) to represent major bioengineering factors. Both mesenchymal cells (HSCs and MSCs) accelerated organoid growth and promoted spontaneous complex liver-like microtissue (BLT) formation. DS and PIC further improved either BLT formation or functionality. Transcriptomic analyses revealed the integrated cellular and extracellular cues in BLT formation and maintenance. To conclude, organoids augmented with mesenchymal cells in chemically defined hydrogels yield functional BLTs suitable for disease modelling, drug screening, and toxicity tests, and form an important basis for future development of larger liver tissues for *in vivo* transplantation. The bioengineering strategy developed in this study can be extended to engineer other types of tissues and also be utilized to investigate the interaction of different bioengineering factors.

## Highlights

• Mesenchymal cells enhance the expansion of organoids.
• Dynamic suspension culture promotes tissue formation and hepatic differentiation.
• Chemically defined PIC-Collagen hydrogels enhance bioengineered tissue maturation.
• Transcriptomics reveal enhanced extracellular matrix remodeling by mesenchymal cells.

## Graphical abstract

**Figure:**
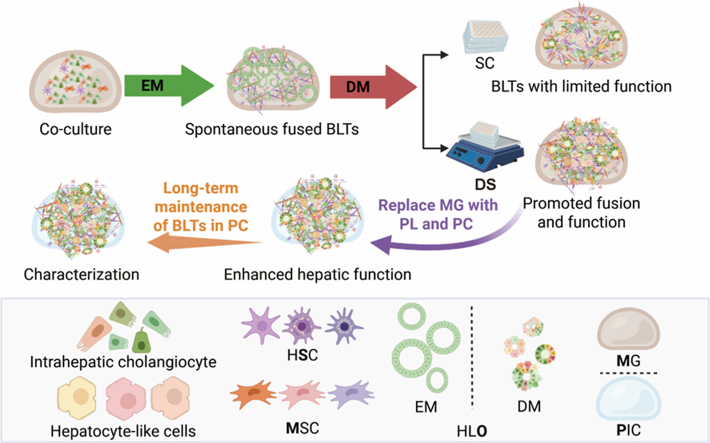

## 1 Introduction

Human organoids have emerged as powerful tools for disease modeling and fundamental biological studies, also as vital cell sources for tissue engineering and regenerative medicine (TERM) [1]. However, current approaches for bioengineered tissues often lack the incorporation of major bioengineering factors (i.e., cellular complexity, well-defined extracellular matrix (ECM) mimicry, dynamic stimuli). This limitation hampers their utility in dissecting the roles of individual influence factors (*e.g.*, cell types and ECM components) in tissue homeostasis, disease progression, and regeneration [2]. A new approach, combining human liver-derived epithelial organoids with non-parenchymal cells and well-defined matrices into bioengineered liver tissues (BLTs), will more faithfully recapitulate native liver function and provide insights into the mechanistic process governing tissue organization and maturation.

The fundamental components of BLTs are functional hepatic cells and supportive biomaterials that function as extracellular matrix (ECM) mimicry. The human liver consists of approximately 70-80% parenchymal cells (mostly hepatocytes and 3-5% cholangiocytes) and 20-30% nonparenchymal cells, mainly including 5-10% mesenchymal cells, 10-15% endothelial cells, around 3% Kupffer cells, and lymphocytes [[3], [4], [5]]. Hepatocytes are the major cell type that performs vital liver functions; however, due to the limited availability of primary human hepatocytes (PHH), current tissue engineering strategies have shifted to stem cell-based cell sources and their subsequent differentiation into hepatocyte-like cells [6,7]. Human intrahepatic cholangiocyte organoids (ICOs) mainly consist of adult liver stem cells, can be expanded for a long time into a large number of cells with stable genomic profiles, and can also be differentiated into hepatocyte- and cholangiocyte-like cells *in vitro*, making them suitable for LTE [[8], [9], [10]]. However, since ICOs consist of epithelial cells only, they are far from mimicking the complexity and function of the liver. For example, mesenchymal cells, including liver mesenchymal stromal cells (MSCs) and hepatic stellate cells (HSCs), play important roles in liver regeneration and directly impact liver function [11,12]. They secrete growth factors, such as hepatocyte growth factor (HGF), epidermal growth factor (EGF), fibroblast growth factor (FGF), transforming growth factor-beta (TGFβ), and bone morphogenetic protein (BMP), produce and remodel the ECM, and regulate the stem cell niche [3,13]. All these factors influence parenchymal cells in liver homeostasis, as well as in liver regeneration. Importantly, mesenchymal cells also enable tissue formation *in vitro* and improve engraftment after transplantation [14,15]. Here, we evaluated the use of bi-potential epithelial organoids (ICOs, hereafter organoids) and mesenchymal cells (HSCs and MSCs) for the generation of BLTs.

Various hydrogels have been developed to mimic the ECM for TERM approaches, both *in vitro* and *in vivo* [16,17]. For organoid culture, Matrigel is currently the gold standard [18], primarily due to its complex biological composition, which supports organoid formation and proliferation. Due to shortcomings of Matrigel, such as being animal-derived, ill-defined, and with large batch-to-batch variations, scientists have developed (semi-)synthetic hydrogels as Matrigel alternatives, although the biological complexity of Matrigel is hard to mimic [16,17]. Among those (semi-)synthetic hydrogels, polyisocyanide-based (PIC) hydrogels are drawing increasing attention in the field of TERM [19,20]. PIC is synthetic and thermoreversible and was originally developed to mimic the porous and fibrous architecture and mechanical properties of ECM proteins, such as collagen and fibrin [[21], [22], [23]]. With these advantages, PIC hydrogels have been successfully used for organoid culture *in vitro* [19,20] and for *in vivo* wound healing studies [24,25]. However, PIC has no bioactive binding sites for cell attachment and downstream signaling. Since collagen-I has been widely used in the TERM field and was recently reported to improve the hepatic functions of hepatocyte spheroids [26], we combined PIC with collagen-I for BLT generation.

In this study, we bioengineered liver tissues by co-culturing human liver epithelial organoids with mesenchymal cells, including HSCs and MSCs. We cultured BLTs on an orbital shaker to create a dynamic microenvironment. Dynamic suspension (DS) culture methods have been previously reported to accelerate the growth of human epithelial organoids [10,27] and/or promote the maturation of organoids [[28], [29], [30]]. In our co-cultures, the DS method enhanced spontaneous tissue formation. Subsequently, we replaced Matrigel with a well-defined hydrogel for the co-culture, using the synthetic, thermoreversible PIC supplemented with collagen-I. We investigated tissue formation, cellular composition, morphology, and maturation of the BLTs in all conditions. Further, we conducted transcriptomic analyses of BLTs and propose a model explaining the contributions of cellular and extracellular factors to tissue formation and maturation. As such, this study provides a straightforward method for bioengineering complex human liver tissues that can be directly applied for studying cell-cell and cell-matrix interactions in homeostasis, disease and regeneration.

## 2 Results

### 2.1 Mesenchymal cells enhance the expansion of organoids

**Fig. 1:**
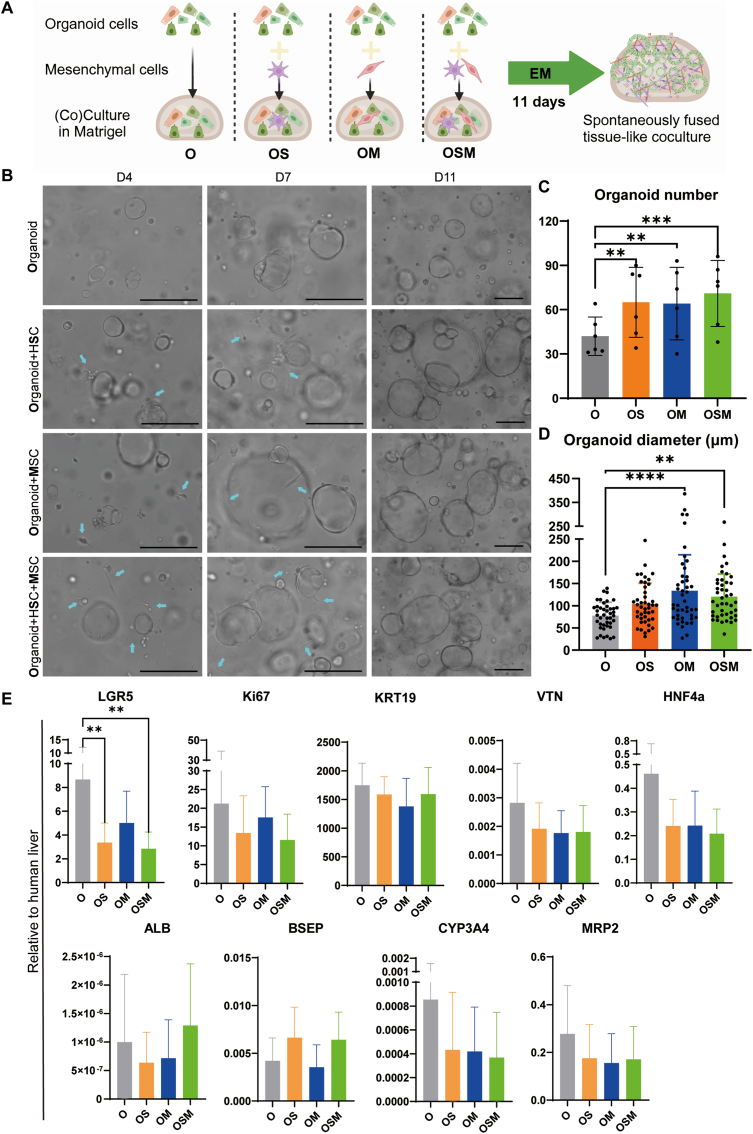
Expansion of co-cultures in Matrigel. n = 6. (A) Schematic of different cellular compositions in Matrigel droplets. O for organoid, S for hepatic stellate cell (HSC), and M for mesenchymal stem cell (MSC). (B) Bright-field pictures showing the morphology of organoids co-cultured with or without HSCs and MSCs in Matrigel in expansion medium (EM) at three time points, D4 (left), D7 (middle), and D11 (right). Organoids only (O), organoids with HSCs (OS), organoids with MSCs (OM), organoids with HSCs and MSCs (OSM). Arrows indicate mesenchymal cells (MSC or HSC). Scale bar = 200 μm (C) Organoid number quantification. Organoid numbers were quantified from 6 donors and at least 3 pictures per donor. (D) Organoid diameter quantification and distribution. Organoid diameters were measured from 6 donors and at least 10 organoids per donor. (E) Gene expression of co-cultures expanded in Matrigel for 11 days, compared to human liver tissues. Stem cell/progenitor marker LGR5, proliferative marker Ki67, ductal marker KRT19, and hepatocyte markers HNF4a, ALB, CYP3A4, BSEP, MRP2, and VTN were used for the qPCR assays. Significant analysis was labeled with ∗ (p < 0.05), ∗∗ (p < 0.01), and ∗∗∗ (p < 0.001).

The human liver consists of multiple types of cells, and missing any cell type leads to liver dysfunction. To mimic this cellular complexity in bioengineered human liver tissues (BLTs), we co-cultured organoids with HSCs and MSCs in expansion medium (EM) at a 6:1:1 ratio (Fig. 1A). Since organoids reach a 4-5 fold expansion within one week, this cell ratio should result in a final percentage of approximately 90% epithelial cells and 6-8% mesenchymal cells, mimicking *in vivo* cell ratios [[3], [4], [5]]. We observed that all co-cultures formed more and larger organoids than the control group with organoids only 4-7 days (D4-D7) after single-cell seeding in Matrigel (Fig. 1B). We confirmed this observation by quantification of organoid numbers (Fig. 1C) and organoid diameters (Fig. 1D). On day 11 (D11), the co-cultures were almost confluent while organoids in the control group were sparser (Fig. 1B). When we characterized the co-cultures with qPCR assays, we found that the stem cell/progenitor marker *LGR5* was significantly lower in co-cultures containing HSCs (Fig. 1E). Hepatocyte markers, including *HNF4A*, *ALB*, and *CYP3A4,* indicated that all cultures expressed the markers at similar levels, which were much lower compared to liver tissue, in line with expansion conditions. Importantly, the expression of the ductal marker *KRT19* was comparable among all groups, indicating that HSCs and MSCs did not hamper the ductal phenotype of organoids cultured in EM. Instead, the mesenchymal cells enhanced the growth of organoids in EM. Controls containing only mesenchymal cells (both HSCs and MSCs) showed no obvious proliferation till D11 in EM (Fig. S1A) and very low expression of hepatocyte marker genes, except for *BSEP* (Fig. S1B).

### 2.2 HSCs, but not MSCs, hamper the differentiation of organoids

**Fig. 2:**
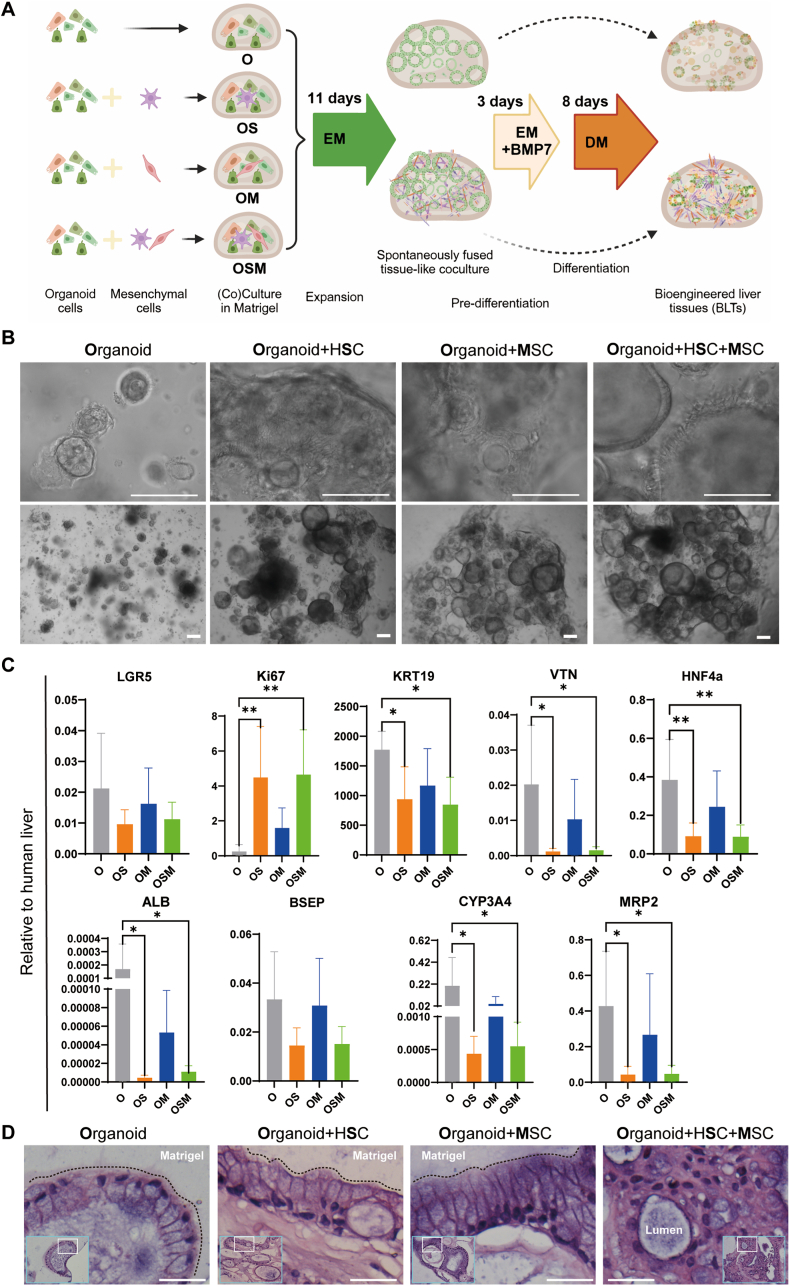
Differentiation of co-cultures in Matrigel under static culture (SC) conditions. n = 6. (A) Schematic of different cellular compositions as co-cultures from EM to differentiation medium (DM). (B) Bright-field pictures showing the morphology of organoids co-cultured with HSC and/or MSCs in Matrigel with DM for 8 days at two magnifications, zoomed-in (top) and overall view (bottom). Scale bar = 200 μm (C) Gene expression of co-cultures differentiated in Matrigel for 8 days, compared to human liver tissues. Primers of the same makers as in EM were used for the qPCR assays. Significant analysis is labeled with ∗ (p < 0.05) and ∗∗ (p < 0.01). (D) H&E staining images showing the morphology of organoids co-cultured with HSC and/or MSCs in Matrigel with DM for 8 days. Scale bar = 10 μm.

After establishing the co-cultures of organoids with HSCs and/or MSCs in expansion medium, we continued to induce hepatic differentiation with differentiation medium (DM) when the cultures were confluent (Fig. 2A). After 8 days of differentiation, light microscopy analysis showed that organoids became more condensed and darker compared to EM and all the co-cultures spontaneously assembled into more complex structures (Fig. 2B). Most HSCs and MSCs were not visible anymore as single cells like in EM conditions (Fig. 1B), indicating that they may have fused with organoids. Organoids that had fused with surrounding cells seemed to have opened their lumen; however, this spontaneous fusion was not complete due to partial attachment of cells to the standard culture plates. In control groups containing only mesenchymal cells, we observed enhanced proliferation compared to EM conditions, with clear cellular interactions and MSC cluster formation (Fig. S2A). Compared to the expression levels of EM conditions (Fig. 1C), most hepatocyte markers (*ALB*, *CYP3A4*, and *BSEP*) were upregulated in all conditions after differentiation, while the stem cell marker *LGR5* and the proliferation marker *Ki67* were downregulated (Fig. 2C). Hepatocyte markers were comparably expressed in organoid-only culture and the co-culture of organoids with MSCs, but the expression of most markers in co-cultures containing HSCs was significantly lower (Fig. 2C). In contrast, the expression levels of *Ki67* were significantly higher in co-cultures containing HSCs. Once mesenchymal cell-only controls were included in the graphs, we found that the expression levels of *LGR5* and *Ki67* in mesenchymal cells were upregulated in DM compared to their expression in EM, particularly in HSCs, while hepatocyte markers remained low in mesenchymal cells (Fig. S2B). We further compared the different co-cultures with histological analysis by H&E staining. H&E results showed that all cocultured conditions displayed more complex structures than the organoid-only condition, with the tri-culture (OSM) being the most compact (Fig. 2D). Since the co-culture condition of organoids with HSCs and MSCs resulted in efficient tissue formation and most closely reflects the cellular complexity of the liver, we continued with this OSM condition for our further studies.

### 2.3 Dynamic suspension culture promotes BLT formation and hepatic differentiation

**Fig. 3:**
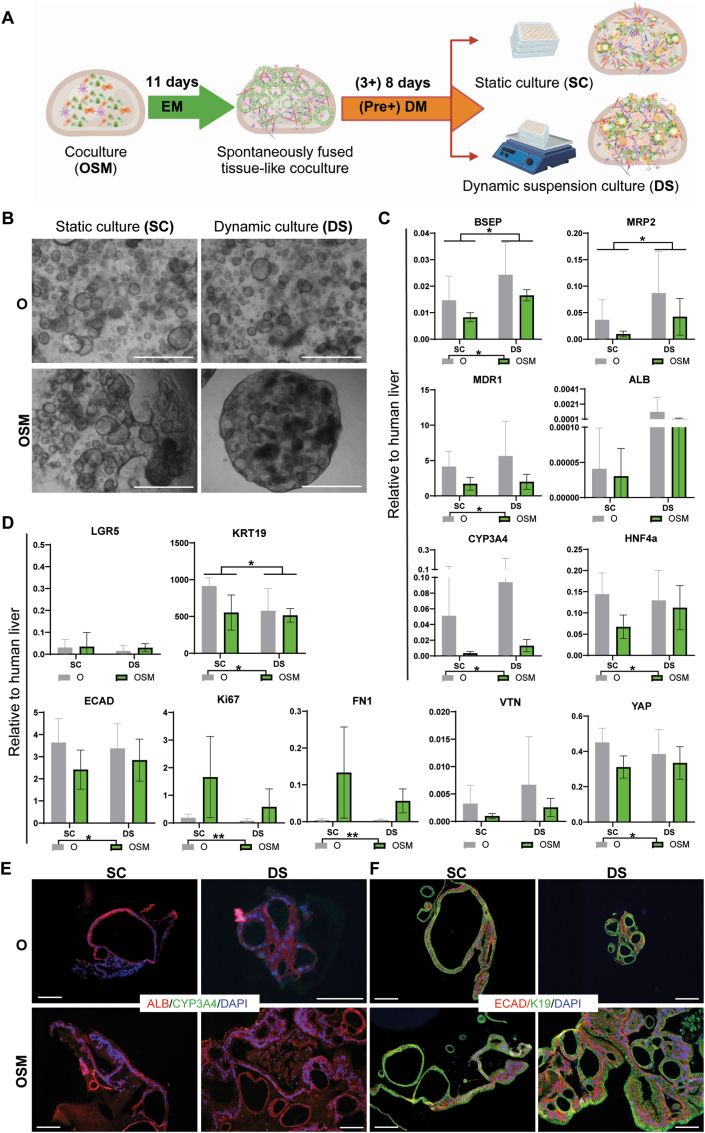
Characterization of BLTs in static or dynamic conditions. n = 6. (A) Schematic of the experimental setup comparing static culture (SC) and dynamic suspension culture (DS) conditions. (B) Morphology of organoid-only (O) and co-cultures (OSM) derived bioengineered liver tissues (BLTs) in SC or DS conditions after 8 days of differentiation. DS meant the cells were plated in Matrigel droplets in low-attachment plates which were put on a horizontal shaker at a speed of 70 rpm. Scale bar = 1000 μm. (C-D) Gene expression of BLTs in SC vs DS conditions, compared to human liver tissues. Besides the markers previously used for SC characterization, more markers were used for the qPCR assays, including epithelial marker ECAD, polarity marker MDR1, ECM component FN, and mechanotransduction marker YAP. Significant analysis was labeled with ∗ (p < 0.05) and ∗∗ (p < 0.01). Statistical significant labels for SC vs DS or O vs OSM were shown above or below the bars, respectively. (E-F) Characterization of BLTs by immunofluorescent (IF) staining. Functional hepatocyte proteins ALB (red) and CYP3A4 (green); Epithelial protein ECAD (red), and ductal protein KRT19 (K19, green). Scale bar = 100 μm.

To avoid attachment of cells to the bottom of the culture plates and enhance the spontaneous tissue formation, we next utilized low-attachment plates and transferred the plates to an orbital shaker for dynamic suspension culture (DS) at 70 rpm during the differentiation process (Fig. 3A), in line with our previous observation that a rotational speed of 60-80 rpm in a stirred suspension culture resulted in optimized organoid expansion and differentiation [10,27]. Bright-field pictures showed that the OSM-derived BLTs fused more than organoid-only derived BLTs in both static culture (SC) and DS. In addition, the OSM condition formed more compact tissues in DS conditions compared to SC (Fig. 3B). Then, we compared the differentiation of all cultures by qPCR. Most hepatocyte markers (*ALB*, *CYP3A4*, *BSEP*, *MRP2*, and *MDR1)* showed a (trend of) higher expression in DS than in SC in organoid-only as well as OSM conditions (Fig. 3C). Consistent with the previous results when differentiated in normal adhesion plates (Fig. 2), BLTs derived from both OSM and organoid-only showed downregulation of stem cell marker *LGR5* and proliferation marker *Ki67* after differentiation compared to EM conditions (Fig. 3D). Also, *Ki67* was significantly higher in BLTs derived from OSM than from organoid-only, along with a lower expression of the majority of hepatocyte markers (*CYP3A4*, *BSEP*, *KRT19*, *ECAD*, *HNF4a*, and *MDR1*) (Fig. 3C and D). Interestingly, the extracellular matrix (ECM) component genes fibronectin (*FN*) and vitronectin (*VTN*) showed opposite trends of expression between organoid-only and OSM-derived BLTs, with *FN* higher in OSM and *VTN* higher in organoid-only cultures (Fig. 3D). Moreover, the mechanotransduction marker *YAP* was significantly lower expressed in OSM than in organoid-only cultures, and the expression levels of *YAP* were comparable between SC and DS (Fig. 3D), indicating differential expression of *YAP* in different cell types rather than differences in culture conditions. We further verified the hepatic and epithelial phenotype of both OSM and organoid-only derived BLTs on the protein level with immunofluorescent (IF) staining. IF results showed that all BLTs expressed Albumin (ALB) after differentiation, while no specific signal for CYP3A4 was observed (Fig. 3E and Fig. S3A). The epithelial protein E-cadherin (ECAD) and the ductal protein KRT19 (K19) were both expressed in all conditions (Fig. 3F).

### 2.4 PIC-collagen hydrogels enhance the maturation of BLTs

**Fig. 4:**
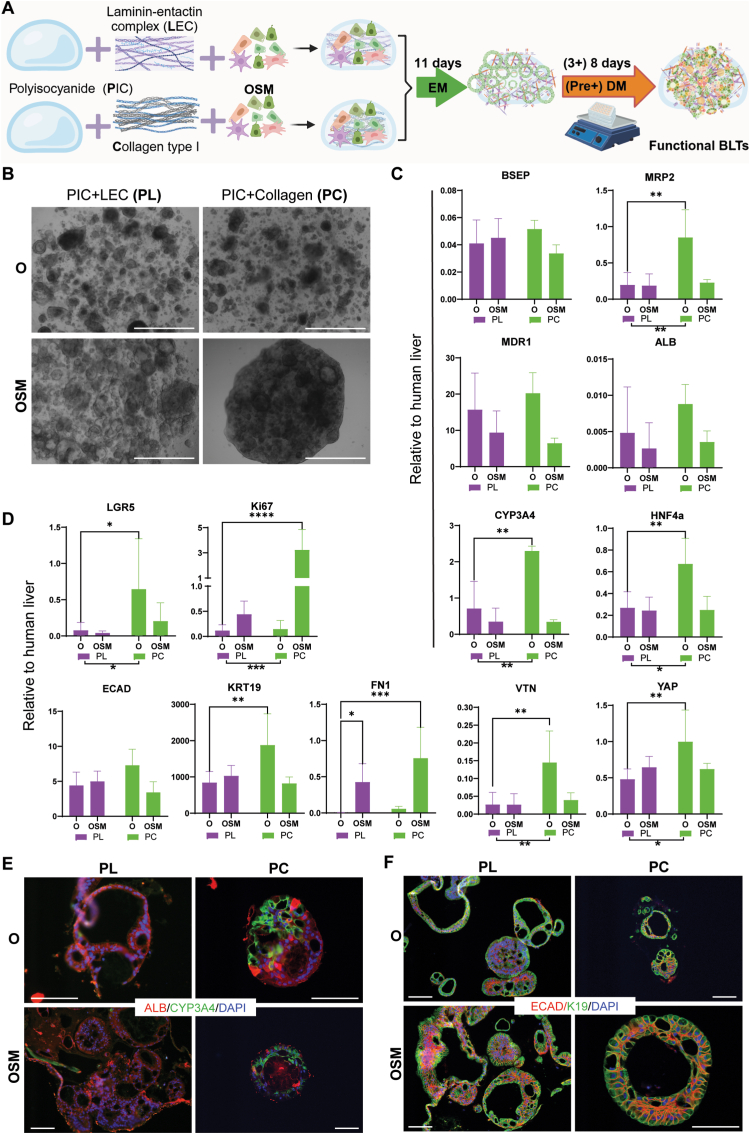
Characterization of BLTs formed in PIC hydrogels. n = 3. (A) Schematic of co-cultures (OSM) in polyisocyanide (PIC)-based hydrogels for bioengineered liver tissues (BLTs). (B) Morphology of BLTs in PIC hydrogels after 8 days of differentiation. PL = PIC + Laminin-entactin complex (LEC); PC = PIC + Collagen-I. Scale bar = 1000 μm. (C-D) Gene expression of BLTs differentiated for 8 days in PIC hydrogels, compared to human liver tissues. The same markers were used as for characterizing samples in Matrigel under DS conditions. Significant analysis was labeled with ∗ (p < 0.05), ∗∗ (p < 0.01), and ∗∗∗ (p < 0.001). Statistical significant labels between individual bars were shown above the bars and labels for PL vs PC were shown below the bars. (E-F) Characterization of BLTs by IF staining. (E) Functional hepatocyte proteins ALB (red) and CYP3A4 (green); (F) Epithelial protein ECAD (red) and ductal protein KRT19 (green). Scale bar = 100 μm.

The co-culture of organoids with HSCs and MSCs formed fused liver tissues (BLTs) in Matrigel droplets with liver functionality after differentiation. However, Matrigel has many well-known disadvantages, such as large batch-to-batch variation and its mouse tumor origin, which make it unsuitable for clinical applications. In addition, we hypothesized that the immature nature of our BLTs was partly caused by the abundant and diverse ECM components in Matrigel that may maintain a stem cell niche and prevent further differentiation. Modulation of the microenvironment was shown to improve engraftment and function after transplantation [7]. Hence, we developed polyisocyanide (PIC)-based hybrid-hydrogels to bioengineer liver tissue, supplemented with two major ECM proteins, either laminin-entactin complex (LEC) or collagen-I (Fig. 4A). Previously, we reported that PIC-LEC (PL) hydrogel was sufficient for the expansion of organoids, whereas organoid differentiation towards hepatocytes in PL was incomplete, comparable to Matrigel [19]. Here, we supplemented PIC with collagen-I (PC) to enhance hepatic differentiation [26]. Consistent with the morphology in Matrigel droplets, OSM fused more than organoid-only culture and formed compact BLTs with the DS method, especially in the PC hydrogel (Fig. 4B). Gene expression analysis by qPCR assays showed that most hepatocyte markers were significantly higher expressed in PIC hydrogels, particularly in PC, compared to Matrigel (Fig. S3B). Then, we compared the differentiation of BLTs derived from organoid-only and OSM in PL and PC, respectively. The results showed that some of the hepatic markers analyzed (*ALB*, *BSEP*, and *MDR1*) were comparably expressed in PL and PC, whereas others (*CYP3A4*, *MRP2*, and *HNF4a*) were significantly upregulated in PC compared to PL (Fig. 4C). Interestingly, the stem cell marker *LGR5* and proliferation marker *Ki67* were significantly higher expressed in PC than in PL, just as the ductal marker *KRT19*, ECM components *FN* and *VTN*, and mechanotransduction marker *YAP* (Fig. 4D). The epithelial marker *ECAD* was well maintained in all conditions. We further verified the expression of hepatic markers on the protein level by IF staining. The results confirmed the presence of both ALB and CYP3A4 proteins (Fig. 4E and Fig. S3C). Notably, CYP3A4 was rarely detected at protein level in BLTs differentiated in Matrigel droplets (Fig. 3E and Fig. S3A). Epithelial protein ECAD and ductal protein KRT19 were both detected in all BLTs (Fig. 4F). In summary, we showed that PIC hydrogels are suitable to replace Matrigel for liver tissue engineering using co-cultures (OSM) of organoids with HSCs and MSCs, and supplementation of PIC with collagen-I improved fusion and hepatic maturation of the BLTs.

### 2.5 BLTs retain functional hepatic characteristics for over two weeks

**Fig. 5:**
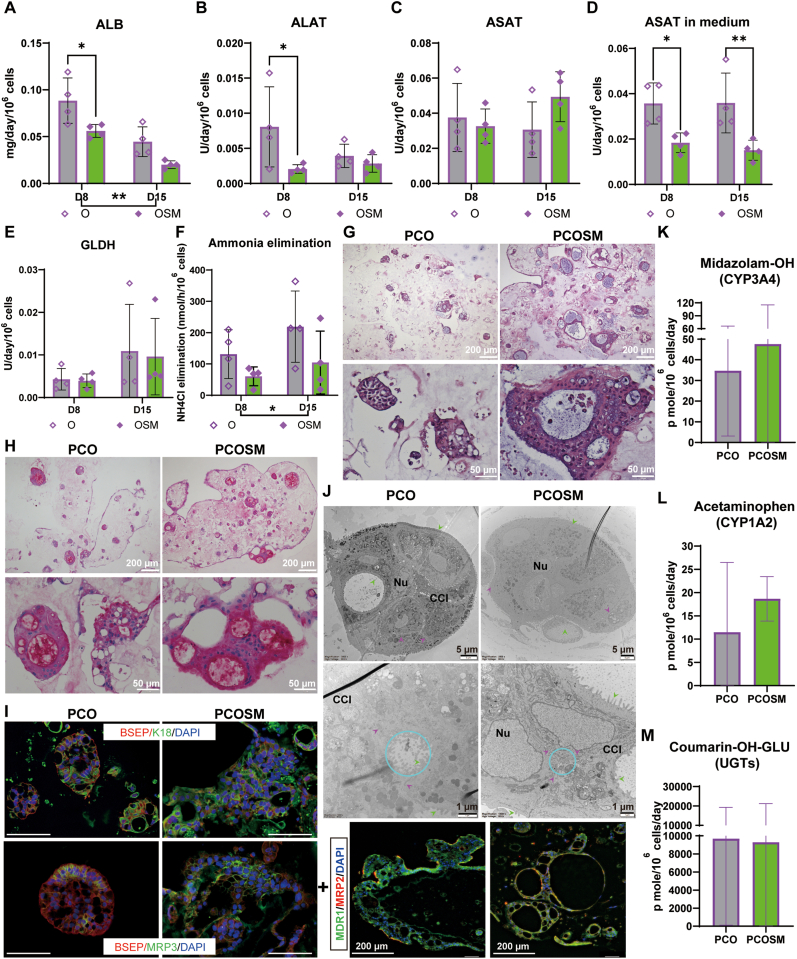
BLTs retain functional hepatic characteristics for over two weeks. (A-C) Intracellular protein levels of ALB, ALAT, ASAT. (D) ASAT levels in the medium. (E) Intracellular levels of GLDH. (F) Ammonia elimination assay to assess the ammonia detoxification capacity of bioengineered liver tissues (BLTs). The assessment of protein levels was measured with medium incubated with cells for 24 h. The BLTs for ammonia elimination assays were incubated with DM with the addition of NH4Cl for 24 h. Both protein levels and ammonia elimination assays were assessed at two timepoints (i.e. D8 and D15). Significant analysis was labeled with ∗ (p < 0.05) and ∗∗ (p < 0.01). Statistical significant labels between individual bars were shown above the bars and labels for D8 vs D15 were shown below the bars. (G) H&E staining and (H) PAS staining of BLTs. (I) IF staining with apical marker BSEP in combination with hepatocyte marker keratin 18 (K18, upper) or with basolateral marker MRP3 (lower), and apical markers MDR1 and MRP2 on the right side. Scale bar = 100 μm. (J) Subcellular characterization of BLTs with TEM analysis. Nucleus (Nu), bile canaliculi-like structures (blue circle), tight junction (purple arrow), microvilli (green arrow), and cell-cell interactions (CCI). (K-M) Compounds assays to determine metabolizing capabilities of BLTs. Metabolite formation was measured by LC-MC/MS and normalized to live cell numbers, reflecting the enzymatic activities of phase I (CYP3A4, CYP1A2) and phase II (UGTs), displayed in (K), (L), (M), respectively. n = 4.

We further investigated the hepatic functions of the BLTs with PC hydrogels after 15 days of maintenance in dynamic suspension (DS). These include intracellular protein levels of ALB, ALAT, ASAT, GLDH, and ASAT in the culture medium (Fig. 5A–E). We observed an overall decrease in ALB level from D8 to D15 for both organoid-only (O) and co-culture (OSM) conditions. Interestingly, there was no significant difference between O and OSM conditions anymore at D15 (Fig. 5A). ALAT levels remained similar between D8 and D15, and again, we did not observe a significant difference between O and OSM conditions anymore at D15 (Fig. 5B). The intracellular levels of ASAT between O and OSM were similar, as between D8 and D15 (Fig. 5C). Interestingly, ASAT levels in the medium were always lower in OSM than O, with no difference between D8 and D15, indicating that there might be less cell damage in the OSM condition (Fig. 5D). Contrary to the decreased ALB levels, there was a trend of increasing GLDH levels from D8 to D15, with no difference between O and OSM (Fig. 5E). Consistent with intracellular GLDH levels, the ammonia elimination ability of BLTs increased from D8 to D15 with comparable levels between O and OSM conditions (Fig. 5F). We then characterized the BLTs with histological analyses after 15 days of differentiation. H&E images show that there were overall more and bigger fused structures in OSM than in O (Fig. 5G). Both O- and OSM-formed BLTs displayed remarkable glycogen storage, as determined by PAS staining (Fig. 5H). Notably, organoids in Matrigel have a clear polarity with the apical side in the lumen and the basal side facing the matrix. However, based on the location of the nucleus and glycogen from the H&E and PAS stainings, we found that the polarity of organoids in the BLTs in PC could have changed. Therefore, we conducted IF staining with hepatocyte marker keratin 18 (K18), apical markers BSEP, MDR1 and MRP2, and basolateral marker MRP3. The results show that the apical protein BSEP is located mostly on the outside of organoids in PC with clear presence of K18 and MRP3, which is strongly expressed in certain locations of the BLTs (Fig. 5I). Quantification of IF stainings for the apical marker Villin-1 (VIL) indicated that OSM obtained an overall mixed polarity in both Matrigel and PC hydrogels while O showed a predominant apical-out polarity in PC hydrogels instead of the conventional apical-in polarity seen in Matrigel (Fig. S3G–J). Further, we investigated the subcellular structures of BLTs with transmission electron microscopy (TEM) analysis. The TEM images showed the presence of tight junctions, bile canaliculi-like structures, and microvilli in both O- and OSM-derived BLTs in PC (Fig. 5J). Compared to BLTs in Matrigel (Fig. S3D), BLTs in PC formed more tight junctions and bile canaliculi-like structures, and their microvilli were located both outside of the organoids/BLTs and inside the lumens (Fig. 5J), which is another sign of mixed polarity. The number of microvilli per nanometer (nm) membrane were comparable among different conditions (Fig. S3E), the number of bile canaliculi-like structures and the lengths of tight junctions were also mostly comparable, except for the MGO condition, which had longer tight junctions (Fig. S3F). Furthermore, we treated BLTs with a cocktail of substrates of phase I (CYP3A4, CYP1A2) and phase II (UGTs) enzymes and measured the parent compound depletion and metabolite formation with LC-MS/MS. The results show that both O- and OSM-derived BLTs in PC were able to perform comparable drug metabolizing capabilities after 15 days in culture (Fig. 5K–M). Taken together, BLTs in PC formed structures closer mimicking natural liver than in Matrigel and retained key hepatic functions after 15 days of culture.

**Fig. 6:**
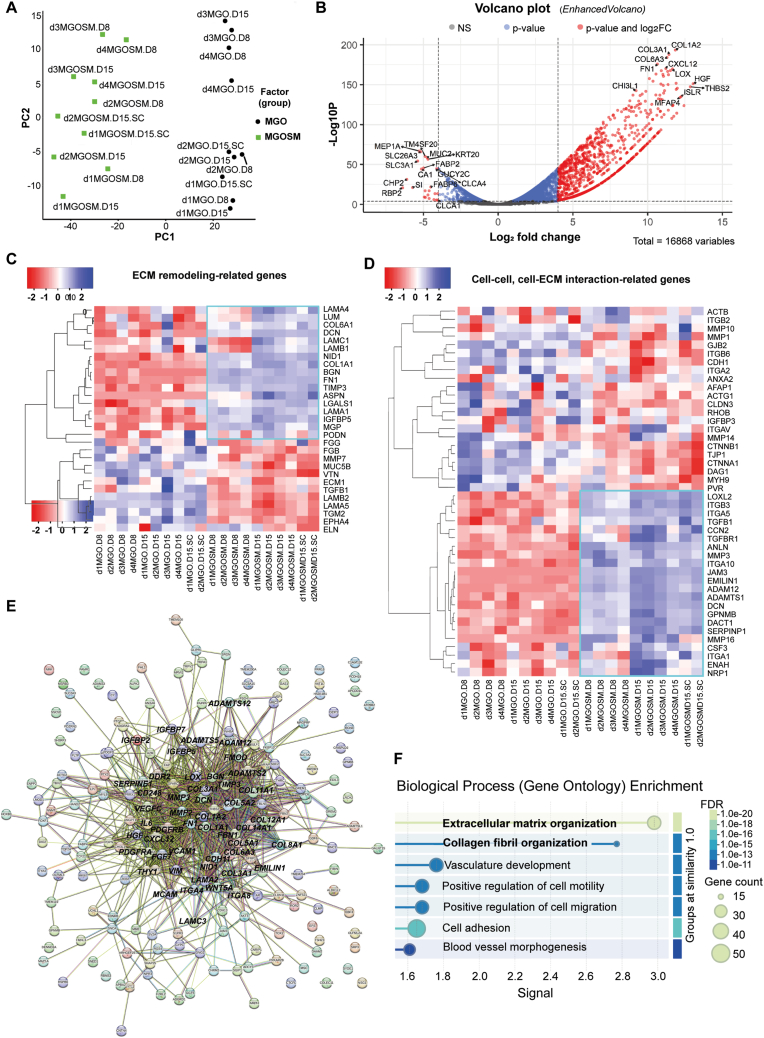
Transcriptomics reveal enhanced ECM remodeling by mesenchymal cells. (A) PCA graph shows the contribution of the top two principal components to the variance in mRNA expression between the organoid-only (O) and co-culture (OSM) conditions in Matrigel (MG). The relation between the top two principal components (PC1 and PC2) highlights the separation of the MGO and MGOSM samples. (B) Volcano plots showing log2 fold change (FC) versus significance (p-value) calculated for 16868 genes for the two independent populations, highlighting enriched genes for the MGO and MGOSM conditions. Dots in red indicate log2FC < -4 or >4, and with adjusted p-value <0.0001. Heatmaps were made out of overlapped genes between the 16868 genes and previously reported genes that are associated with ECM-remodeling (C), cell-cell & cell-ECM interaction (D). (E) Interaction networks of the top 200 most differently expressed genes between O and OSM. (F) Gene Ontology analysis reveals the top involved signaling pathways for the top 200 differently expressed genes between O and OSM. n = 4.

**Fig. 7:**
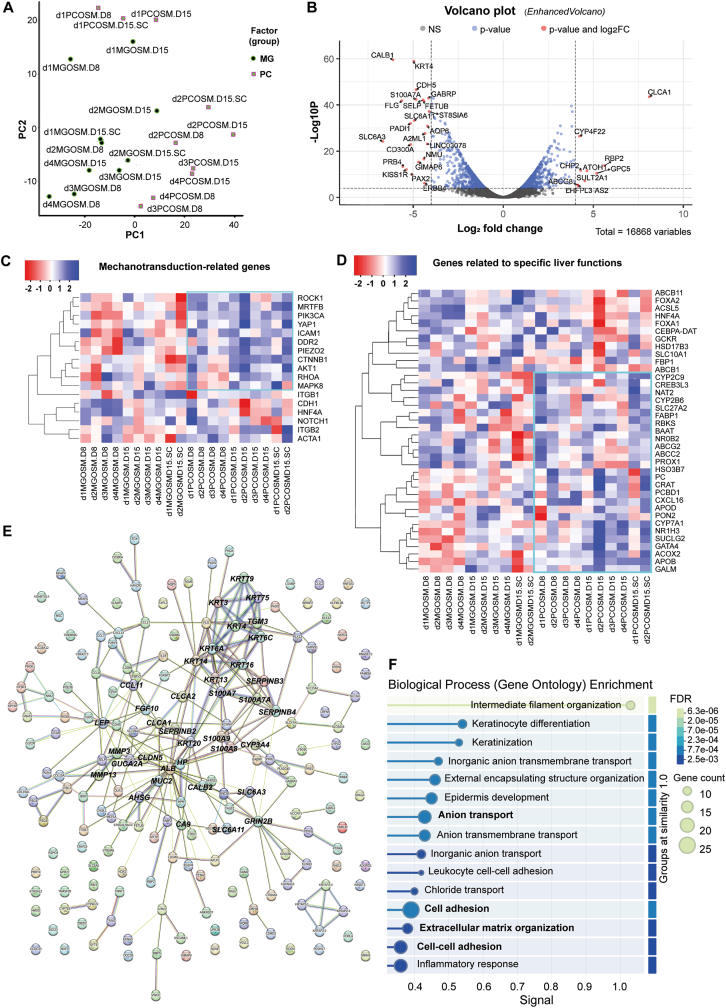
Transcriptomic comparison of BLTs in Matrigel and PC. (A) PCA graph shows the contribution of the top two principal components to the variance in mRNA expression between the Matrigel (MG) and PIC + Collagen (PC) conditions for co-cultures with OSM to form BLTs. The relation between the top two principal components (PC1 and PC2) highlights the separation of the MGOSM and PCOSM samples. (B) Volcano plots showing log2 fold change versus significance (p-value) calculated for all genes for the two independent populations, highlighting enriched genes for the MGOSM and PCOSM conditions. Dots in red indicate log2FC < -4 or >4, and with adjusted p-value <0.0001. (C-D) Heatmaps were made out of genes reported to play a role in mechanotransduction, or specific liver functions. (E) Interaction networks of the top 200 most differently expressed genes between MG and PC conditions. (F) GO analysis reveals the top involved signaling pathways for the top 200 differently expressed genes between MG and PC conditions. n = 4.

To better understand the molecular mechanisms driving tissue formation and maturation in the different culture conditions we used, we conducted bulk RNA-seq analyses. Principal component analysis (PCA) displays clear clustering of organoid-only (O) and co-culture (OSM) conditions (Fig. S4A). Within OSM conditions, samples formed several small subgroups mainly based on hydrogels - Matrigel (MG) or PIC + Collagen (PC) - and donors (d1, d2, d3, d4). While, the differences between static culture (SC) and dynamic suspension (DS) conditions or between differentiation times (D8 vs D15) are even less clear than donor variations. Consistent with the PCA graph, Pearson correlation analysis shows similar clustering of all samples used for bulk RNA-seq (Fig. S4B). To further investigate the major influences of different culture conditions, we conducted paired analysis within each condition, such as O vs OSM (Fig. 6, Fig. S5), MG vs PC (Fig. 7, Fig. S6), SC vs DS (Fig. S7), and D8 vs D15 (Fig. S8 for O and Fig. S9 for OSM, respectively). To ensure the paired analysis did not introduce any donor-bias, we also performed donor-paired analysis, showing that there were no major differences between the two analysis methods (Fig. S10).

### 2.6 Transcriptomics reveal enhanced ECM remodeling by mesenchymal cells

The PCA graph displays clear clusters of O (right) and OSM (left) conditions (Fig. 6A). Differential expression analysis, shown in a volcano plot, reveals the upregulation of various ECM-related genes in OSM, especially multiple types of collagens, including *COL1A2*, *COL3A1*, *COL6A3* (Fig. 6B). Importantly, hepatocyte growth factor (*HGF*) is among the highest upregulated genes, which could be an explanation for the faster organoid formation and growth in OSM than in O condition (Fig. 1B). Compared to O condition, OSM expressed lower *SLC26A3*, *RBP2*, *FABP2*, *FABP6*, *MUC2*, *CLCA1*, *CLCA4*. Gene ontology (GO) analysis provides information on significant differences in biological processes between O and OSM. The involved activated processes include “collagen-containing extracellular matrix”, “extracellular matrix”, “extracellular matrix organization”. (Fig. S5A left). While the suppressed biological processes are mainly related to the MHC class II protein complex, which may well result from mesenchymal cells (Fig. S5A right). The gene set enrichment analysis (GSEA) shows that the representative GO signaling pathways, including “collagen-containing extracellular matrix”, “extracellular matrix”, “extracellular matrix organization” were all upregulated, while the MHC class II protein complex signal pathway was suppressed (Fig. S5B). To have a closer look at the ECM remodeling and cell-cell and cell-ECM interaction-related gene expression, we applied the normalized gene counts to overlap with lists of previously reported genes (Table S1). The selected genes, displayed as heatmaps, show clear trends of higher or lower expressed genes in O or OSM (Fig. 6C and D). For example, genes (*LAMA1, LAMB1, LAMC1, NID1, and COL1A1*) for the most abundant ECM components (laminin-entactin complex and collagen) are all highly expressed in OSM conditions (Fig. 6C). In turn, several genes for cell-cell and cell-ECM interaction are also highly expressed in OSM conditions. On the contrary, the other genes highly expressed in O are lowly expressed in OSM (Fig. 6D). In addition, we observed a similar pattern of gene expression between O and OSM for autocrine and paracrine effect-related, proliferation and apoptosis-related, and even for specific liver functions-related genes (Fig. S5C–E). We then selected the top 200 most differentially expressed genes between O and OSM to focus on the most outstanding differences and related biological processes. The gene interaction shows that the core of these top 200 differentially expressed genes is mostly related to ECM remodeling, especially laminin-entactin, various collagens, and MMPs. Other genes near the core of the network are related to “cell adhesion and growth” and “liver regeneration”, including *HGF*, *ITGA8*, *IL-6* (Fig. 6E). GO analysis further verified that the enriched biological processes by these top 200 genes are “extracellular matrix organization”, “collagen fibril organization” and “cell adhesion”, which are important for ECM remodeling (Fig. 6F). Taken together, the transcriptomic results reveal that mesenchymal cells enhanced ECM remodeling in BLTs.

### 2.7 Transcriptomics indicate that PC promotes BLT maturation via enhanced mechanotransduction

We have previously found that PC enhanced the maturation of BLTs (Fig. 4), but the underlying mechanism is not clear. Therefore, we compared the OSM-derived BLTs in MG and PC conditions via transcriptomic analysis. The PCA shows clustering of MG (left) and PC (right) conditions except for donor 1(d1)-PC, which is above d1-MG (Fig. 7A). Upregulated genes in PC are related to metabolism, including *CYP4F22*, and *RBP2*, while inflammatory response-related genes, such as *S100A7A* and *CD300A,* are lower expressed in PC (Fig. 7B). GO analysis revealed that multiple ribosomal subunit-related biological processes are activated in PC, indicating enhanced gene translation and active protein synthesis, including collagen trimer (Fig. S6A left). Interestingly, several epidermis development-related processes are suppressed in PC (Fig. S6A right). Graphs by GSEA show the representative GO signaling pathways, including the activated “Collagen trimer”, “cytosolic large ribosomal subunit”, “regulation of cardiac muscle cell membrane repolarization”, and the suppressed “transmembrane signaling receptor activity” (Fig. S6B). The heatmap shows that many mechanotransduction-related genes are upregulated in PC compared to MG, including *YAP1, PIEZO2, DDR2, CTNNB1* (Fig. 7C). Consistent with previous qPCR results, genes related to specific liver functions show an overall higher expression in PC than in MG. These highly expressed genes cover broad liver functions, including bile synthesis (*NR0B2, BAAT, CYP7A1, HSO3B7, ACOX2*) and bile transport (*ABCC2, ABCG2*), drug metabolism (*CYP2B6, CYP2C9, NAT2*), and metabolic activities related to glucose (*GALM, RBKS, PC, SUCLG2*), fat (*FABP1, CRAT, SLC27A2*), cholesterol (*APOB, APOD, PON2, CXCL16*) (Fig. 7D). Interestingly, a distinct group of transcription factors are upregulated (*NR1H3, PCBD1, GATA4, CREB3L3*), whereas others are downregulated (*FOXA1, FOXA2, CEBPA-DAT, HNF4A*) in PC, which is opposite to the trend in MG (Fig. 7D). As PC is a well-defined hydrogel, the pattern of cell-cell and cell-ECM interaction and ECM remodeling within BLTs with PC may well be different from that with MG. Of note, around half of cell-cell and cell-ECM interaction-related genes are higher expressed in PC while the other half are higher in MG, and the highly expressed genes in PC are further upregulated between day 8 (D8) and day 15 (D15) of differentiation, while the lower expressed genes in PC are downregulated between D8 and D15 (Fig. S6C). This phenomenon indicates a dynamic change of gene expression with time; thus, we later conducted a separate comparison between D8 and D15, focusing on O and OSM, respectively. We further checked the genes associated with ECM remodeling and found that around one-third of these genes are higher expressed in PC and also upregulated from D8 to D15. These upregulated genes are closely related to the laminin-entactin complex (*LAMA1, LAMB1, LAMC1, NID1*) that we previously reported to be important for liver progenitor/stem cell growth (Fig. S6D), which may suggest the maintenance of a stem cell niche in BLTs with mesenchymal cells in PC. On the other hand, genes highly expressed in MG are associated with chondrogenesis (*ASPN*), bone formation (*ECM1, BGN*), and one of the major ECM components *COL1A1* together with the *TIMP3* - an inhibitor of matrix metalloproteinases - (Fig. S6D), which are not ideal for the maintenance of hepatic phenotypes. Next, we utilized the top 200 most differentially expressed genes between MG and PC to focus on the most outstanding differences and related biological processes. The gene interaction shows that there are two cores (*ALB* and keratin family) in the interaction network. Genes highly connected to *ALB* include *CYP3A4, GUCA2A*, and the keratin gene family core connects *KRT4, KRT13, KRT14, KRT3, KRT6A*, etc. (Fig. 7E). The enriched biological processes by these 200 genes include “intermediate filament organization”, which shows the strongest signal, and most of the genes are associated with “cell adhesion”, “anion transport”, “cell-cell adhesion”, “inflammatory response”, and also “ECM organization” (Fig. 7F). It is known that the main function of intermediate filaments is to provide support and structure for cells, so the strong intermediate filament organization indicates enhanced mechanical strength and resistance to shear stress for BLTs in PC. These results indicate that the enhanced mechanotransduction could be the reason for the promoted maturation of BLTs in PC.

### 2.8 Transcriptomics reveal stronger influences of DS on OSM than on O

Clustering of static culture (SC) and dynamic suspension culture (DS) samples in the PCA graph is not clear due to tremendous differences in cellular composition and utilized hydrogels (Fig. S7A). GO analysis displays that the most activated biological processes in the DS condition are associated with biosynthesis or metabolic processes besides “Extracellular matrix” (Fig. S7B, left). Surprisingly, the most suppressed biological processes are related to epidermis development (Fig. S7B, right). As the GO analysis focused on the comparison between SC and DS conditions, the influence of DS on O and OSM remains unknown. To investigate if there are different influences of DS on O and OSM, we made heatmaps as described. Most cell-cell and cell-ECM interaction-related genes are expressed higher in OSM than in O, and their expression levels are even higher under DS than under SC conditions (Fig. S7C). Only the minority of genes associated with cell-cell and cell-ECM interaction are higher expressed in O than OSM, with more pronounced differential expression levels under DS conditions (Fig. S7C). In terms of ECM remodeling-related gene expression, although there are clear clusters of genes highly expressed in O but lower in OSM, or genes highly expressed in OSM but lower in O, the expression levels between SC and DS are comparable within O and OSM conditions, respectively (Fig. S7D). Of note, the overall expression of autocrine and paracrine effect-related genes is lower in OSM under DS conditions, while these genes retain similar expression levels in O under both SC and DS conditions (Fig. S7E). As expected, mechanotransduction-related genes are overall highly expressed under DS conditions, especially higher in OSM than in O (Fig. S7F). Taken together, the DS condition affected biosynthetic and metabolic processes and influenced cell-cell and cell-ECM interactions but not ECM remodeling. Also, DS condition suppressed the autocrine and paracrine effects (mainly contributed by mesenchymal cells) in BLTs. The overall influence of the DS condition on BLTs is stronger on OSM (co-culture) than on O (organoid-only).

### 2.9 Extended functional maintenance relies on both cellular and extracellular stimuli

We found differentially expressed hepatic proteins between D8 to D15 (Fig. 5), and transcriptomic comparison of different hydrogels (MG vs PC) and culture conditions (SC vs DS) also indicated changes of gene expression patterns from D8 to D15 (Fig. 6, Fig. 7, S5-S6) and between O and OSM (Fig. S7). To investigate the major contributing factors for extended maintenance of hepatic functions of BLTs, we further compared BLTs between D8 and D15 within O and OSM conditions, respectively. The PCA for O samples between D8 and D15 displays clear separation of samples by donors (d1, d2, d3, d4) with obvious trends of D8 samples located above D15 samples within each donor (Fig. S8A). Instead, the PCA for OSM displays clustering of samples by hydrogels (PC on the right and MG on the left), in addition to donors (Fig. S9A). GO analysis displays distinguishable activated and suppressed biological processes between O and OSM regarding their comparison between D8 and D15 conditions. Ribosome-related biological processes are activated on D15 compared to D8 for O conditions, indicating upregulated protein synthesis (Fig. S8B, left). However, some biological processes related to hepatic functional maintenance are suppressed on D15 for O conditions, including “Digestion”, “Digestive tract morphogenesis”, and “Blood microparticle” (Fig. S9B, right). Compared to OSM samples on D8, OSM samples on D15 show several suppressed biological processes that are filament-related or related to epithelium maintenance, with counts all less than 50 (Fig. S9B, right). Of note, biological processes related to “Vascular/Blood vessel development” and “Tube morphogenesis” are highly activated (Fig. S9B, left), which may contribute to vascularization and engraftment of the BLTs after *in vivo* transplantation.

We further investigated the genes related to “Cell-cell & cell-ECM interaction”, “ECM remodeling”, “Proliferation & apoptosis”, and “specific liver functions” as described above and displayed them as heatmaps. All heatmaps of O samples show no consistent trend of changes in gene expression between D8 and D15 (Fig. S8C–F). Unlike O samples, OSM samples always show clear gene expression trends between D8 and D15, in all four groups of genes shown in the heatmaps. The majority of genes related to cell-cell & cell-ECM interaction are upregulated from D8 to D15, including *ITGA1, ITGAV, ITGB3, DCN, TGFB1*, and *TGFBR1*; the remaining genes of this group show lower expression levels on D15 than on D8, including *CDH1, ITGB2, CLDN3, PVR* (Fig. S9C). Similar trends of gene expression changes are found in ECM remodeling-related genes, with higher expressed genes downregulated and lower expressed genes upregulated from D8 to D15 (Fig. S9D). In particular, the upregulated genes include *LAMA1, LAMB1, LAMC1, NID1*, and *COL1A1*, and these genes encode the most abundant (>90%) ECM proteins, such as laminin-111, entactin, and collagen. It is known that the laminin-entactin complex is important for liver stem/progenitor cell growth, indicating the possibility of maintaining a stem cell niche in the BLTs, contributing to the extended maintenance of hepatic functions. Although around two thirds of displayed liver function related genes are downregulated from D8 to D15, the higher expressed genes are associated with key liver functions, such as bile synthesis (*CYP7A1, ACOX2, HSD3B7*), cholesterol (*CXCL16, APOD, APOB, PON2*), glucose (*PC, GALM, SUCLG2*), transcription factors (*GATA4, NR1H3*) (Fig. S9E). The downregulation of bile transport-related genes (*ABCB1, ABCB11, ABCC2, ABCG2, SLC10A1*) may be due to the polarity change of organoids in the co-cultures with mesenchymal cells, especially in PC. Moreover, all highly expressed genes related to proliferation and apoptosis are downregulated from D8 to D15, such as *EGFR, DKK1, MELK, TGFBR2, CCNA2*, and *MKI67*, and the other well-known proliferative marker *PCNA* is also shown to be downregulated (Fig. S9F). These results suggest that BLTs are lowly proliferative and are protected from apoptosis till D15. Based on the results comparing D8 to D15 samples, we found that cellular (incorporation of epithelial and mesenchymal cells) and extracellular factors (dynamic ECM mimicry) could perform interactive contributions in the extended structural and functional maintenance of BLTs.

### 2.10 ECM remodeling and extracellular mechanical properties influence mechanotransduction in BLTs

**Fig. 8:**
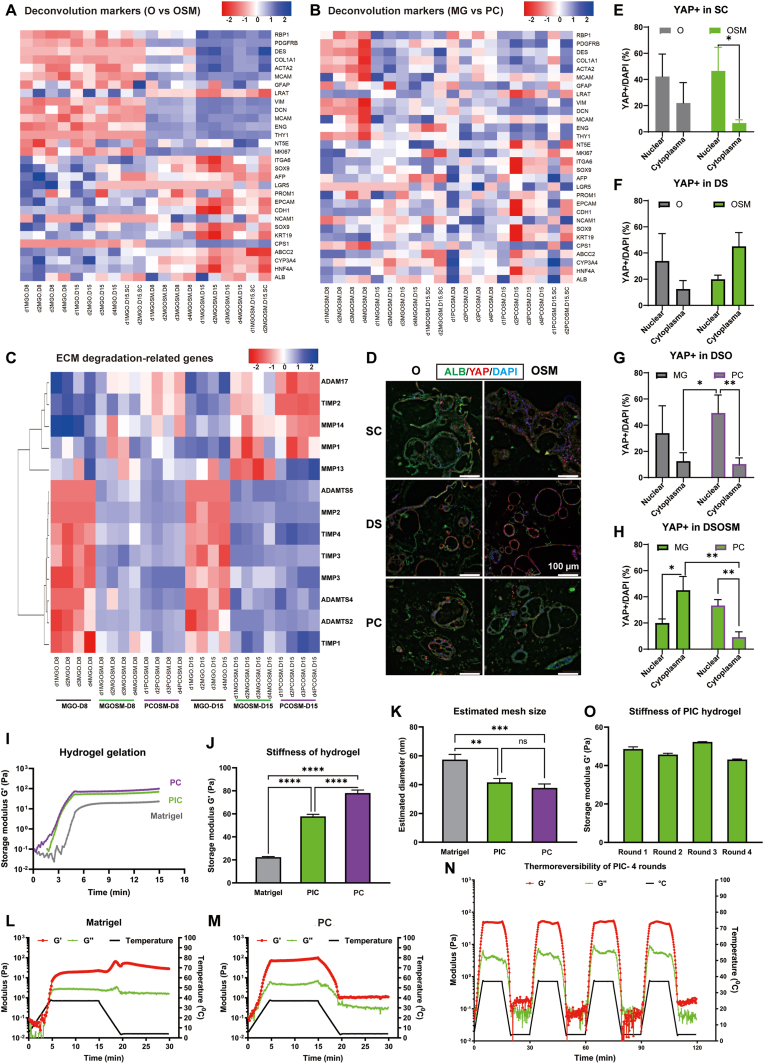
ECM remodeling and extracellular mechanical properties influence mechanotransduction. (A) Deconvolution markers analysis with main focus on the comparison between organoid-only (O) and cocultures (OSM), related to Fig. 6B. (B) Deconvolution markers analysis with main focus on the comparison between Matrigel (MG) and PIC + Collagen I (PC) hydrogels, related to Fig. 7C. (C) ECM degradation-related gene expression on samples from dynamic suspension culture (DS), including the comparison between O vs OSM, MG vs PC, D8 vs D15. (D) IF staining on ALB (green) and YAP (red) for quantification of YAP cellular translocation in O vs OSM under SC or DS culture conditions and in PC hydrogels. Scale bar = 100 μm (E-H) Quantification of YAP localization on BLTs from different culture conditions. (E) static culture (SC) within MG. (F) DS within MG. (G) MG vs PC for O conditions. (H) MG vs PC for OSM conditions. (I) Storage modulus during hydrogel formation. (J) Hydrogel stiffness. (K) Estimated mesh size based on plateau storage modulus. (L) Thermoreversibility of Matrigel. (M) Thermoreversibility of PC. (N) Thermoreversibility of PIC for four rounds of gel/solution transition. (O) Stiffnesses for four rounds PIC gelation. For hydrogel characterizations, n = 3. (For interpretation of the references to colour in this figure legend, the reader is referred to the Web version of this article.)

To further dissect the influence of cell type-specific ECM remodeling and hydrogel properties on mechanotransduction-induced BLT maturation, we first investigated the cell type distribution in BLTs by donor-paired deconvolution analysis. The comparison between O and OSM shows that the overall hepatic markers are higher expressed in O than in OSM and the expression levels show a slight downregulation from D8 to D15 (Fig. 8A), which is consistent with the results shown in Fig. 6. Similarly, mesenchymal markers are overall higher expressed in OSM than in O and show a trend of upregulation from D8 to D15 (Fig. 8A). Interestingly, the expression levels of the urea cycle marker *CPS1* and HSC identity marker *LRAT* display an opposite expression pattern in O and OSM conditions (Fig. 8A). In contrast, when comparing MG with PC conditions, the separation of cell-type specific markers is less obvious than between O and OSM (Fig. 8B). However, the mesenchymal markers are lower expressed in Matrigel than in PC till D8 and mostly upregulated to similar levels by D15. Importantly, there is a trend of slower downregulation of key hepatocyte markers in PC than in Matrigel. Distinctly, *LRAT* expression is downregulated from D8 to D15 while *CPS1* is upregulated from D8 to D15 in both hydrogels (Fig. 8B). These results indicate a predominant role of mesenchymal cells in ECM remodeling, which might be partly attributed to HSC activation in culture. Next, we co-stained mesenchymal marker vimentin (VIM) and proliferative marker Ki67 to characterize the cellular compositions of different BLTs on protein level (Fig. S3K). We quantified the ratios of VIM+, Ki67+ and double-positive cells. The results show that there are overall significant differences in cellular composition between O and OSM regardless of hydrogels (Fig. S3L–M). Within OSM groups, the Ki67+ ratio is always higher than the VIM + Ki67+ ratio, indicating that not only the mesenchymal cells, but also epithelial cells are proliferative in the BLTs (Fig. S3N). The overall VIM + ratio in OSM is around 6-10% (Fig. S3K–M), lower than the initial seeding ratio, in line with an expected around 5-fold organoid expansion before differentiation. We then checked in total 13 ECM remodeling-related genes in the liver and divided them into three major groups: ECM-degrading proteases (mainly MMPs), protease inhibitors (TIMPs) and ECM processing/remodeling enzymes (some ADAMTS). Although the heatmap does not show a clear separation of MMPs and TIMPs or ADAMTSs, the obviously higher expressed genes in O are *ADAM17*, *TIMP2*, *MMP14*, *MMP1* and *MMP13*, while the other genes are higher expressed in OSM, regardless of hydrogels or time (Fig. 8C). The most notable difference between Matrigel and PC is the expression of *MMP13* which is downregulated in Matrigel but highly upregulated in PC, indicating strong ECM breakdown (Fig. 8C). This is in line with the higher expression levels of *LRAT* in PC compared to MG (particularly on day 15) and might hint towards an activation of HSCs in PC conditions during extended culture. We further characterized the expression of the major fibrillar interstitial collagen (COL3A) and the basement membrane protein Laminin by IF staining (Fig. S3O). The quantification results show that both COL3A and Laminin are overall higher expressed in PC than in Matrigel regardless of O or OSM (Fig. S3P–Q). Within the same hydrogel, there is no significant difference of COL3A in both O and OSM (Fig. S3P). However, there is a trend of higher Laminin presence in Matrigel than in PC, particularly in MGOSM (Fig. S3Q), which is mostly likely contributed by the laminins in Matrigel as indicated by the IF images (Fig. S3O). Collectively, these results demonstrate the predominant role of mesenchymal cells in ECM remodeling in the BLTs and indicate an activation of HSCs, particularly in the PC conditions on day 15.

To investigate a mechanistic link between the observed ECM remodeling and upregulation of mechnotransduction pathways in BLTs, we analyzed the intracellular localization of the mechanotransduction marker YAP, which can play important roles in liver organoids as reported [31], together with ALB on the BLTs. The IF images confirmed the presence of ALB and YAP in all conditions (Fig. 8D). In SC, YAP localization is significantly higher in the nucleus than in the cytoplasm (Fig. 8E). In DS, the trend of higher nucleus localization remains in O, but different to SC, there is a trend of higher cytoplasm localization in OSM (Fig. 8F). For organoid-only under dynamic suspension culture (DSO), YAP has a comparable nuclear and cytoplasmic localization in Matrigel but is significantly higher localized in the nucleus in PC (Fig. 8G). In DSOSM, YAP shows opposite cellular localization in Matrigel and PC, with higher cytoplasmic localization in Matrigel but higher nucleic localization in PC (Fig. 8H). These results show an inconsistent influence of DS on O and OSM, and a consistently higher YAP nuclear localization in PC conditions, in line with an upregulation of mechanotransduction pathways in PC conditions (Fig. 6). Importantly, those results might not only be influenced by cell-mediated ECM remodeling, but also by intrinsic hydrogel properties. As such, we further characterized the mechanical properties of the different hydrogels (i.e., Matrigel, PIC, PC). The results show that all three hydrogels showed a similar pattern of gelation during heating from 4°C to 37°C, with PIC samples gelling faster than Matrigel (Fig. 8I). The formed hydrogels displayed significantly differences in stiffness, with PC showing the highest stiffness and Matrigel the lowest (Fig. 8J). Based on the stiffness, we estimated the mesh size of different hydrogels and found that Matrigel had a larger mesh diameter than both PIC and PC (Fig. 8K). As both Matrigel and PIC hydrogels are thermoresponsive, we characterized their thermoreversibility and found that Matrigel formed a gel at 37°C and turned softer at 4°C but did not become fully liquid within the tested time (Fig. 8L). In contrast, both PIC and PC were fully thermoreversible, meaning they formed gels at 37°C and became liquid again at 4°C (Fig. 8M and N). This thermoreversibility was stable for at least four heating-cooling cycles (Fig. 8N) with comparable stiffness across all cycles (Fig. 8O). These results indicate that the enhanced mechanotransduction in PCOSM conditions is most likely a combination of mesenchymal cell induced ECM remodeling and the mechanical hydrogel properties, including increased stiffness and smaller mesh size compared to Matrigel.

## 3 Discussion

In this study, we demonstrate a novel approach to bioengineer liver tissue using a co-culture system of human liver organoids with hepatic stellate cells (HSCs) and mesenchymal stromal/stem cells (MSCs). Liver organoids used in this study are intrahepatic cholangiocyte organoids (ICOs), which consist of epithelial cells that are bipotential and can be differentiated into hepatocyte-like and cholangiocyte-like cells with a respective suitable differentiation medium (DM) [8,32,33]. Hepatocytes and cholangiocytes are the parenchymal cells of the liver, making up ∼80% of the liver cell mass. Here, we combined epithelial organoids with mesenchymal cells (HSCs and MSCs) to induce spontaneous tissue formation and create hepatic tissues representing the cellular complexity of the liver. HSCs are liver mesenchymal cells, well-known for their important roles in liver regeneration [11,34]. MSCs are frequently included in co-culture models to enhance hepatic function in *in vitro* models [35]. Previously, Cordero-Espinoza et al. reported dynamic cell contacts between periportal mesenchyme and ductal epithelium in mouse liver regeneration and murine liver organoids [36]. Recently, Haaker et al. published the results of enhanced proliferation of liver organoids when co-cultured with HSCs [37]. However, to our knowledge, the individual influences of HSCs and MSCs on human organoid expansion, differentiation, and tissue formation remained unknown. In our study, we compared the morphological differences of different organoid cultures: organoids-only (O), organoids co-cultured with HSCs (OS), organoids co-cultured with MSCs (OM), and organoids co-cultured with HSCs and MSCs (OSM). The bright-field pictures showed that all co-cultures resulted in enhanced organoid formation and growth in organoid expansion medium (EM) (Fig. 1B–D), which could be promoted by direct cell-cell contact together with soluble factors secreted by mesenchymal cells as reported previously [36,37]. Our transcriptomic analyses indicate that ECM remodeling by mesenchymal cells could also contribute to the enhanced organoid formation and growth, especially since ECM components like laminin-entactin complex are produced (Fig. 6). Once changed to organoid DM to induce hepatic function, all cultures showed condensed morphologies with obvious differences between organoid-only and co-cultures: in organoid-only conditions, individual organoids adapted a more condensed phenotype, while co-cultures not only became darker than organoid-only, but also spontaneously fused into more compact tissue-like structures, with few visible single mesenchymal cells (Fig. 2B). This spontaneous tissue formation is consistent with the concept of mesenchymal cell-driven iPSC condensation into liver buds reported by Takebe et al. [14]. However, there was no distinguishable morphological difference among OS, OM, and OSM. Therefore, we characterized different cultures on the mRNA level by qPCR assays. The results showed that in EM conditions, the only significant difference was the lower expression of stem cell marker *LGR5* in HSC-containing co-cultures compared to organoids-only (Fig. 1E). In contrast, in DM conditions, the expression levels of most hepatocyte markers were lower in co-cultures containing HSCs, while the expression levels of *Ki67* were higher (Fig. 2C). These results indicate that HSCs could have maintained the organoids in a proliferative state, preventing them from differentiating. However, we cannot exclude that the lower expression of hepatocyte markers (and the trend of downregulation of *LGR5*) could have resulted from the dilution by proliferative HSC mRNA/cDNA, particularly since deconvolution analysis of the RNA sequencing data showed an increase of mesenchymal markers (including HSC markers) over time (Fig. 8A and B). In future studies, the potentially dual role of HSCs during expansion and differentiation of BLTs should be further investigated by single cell RNA sequencing, with special attention to the activation state of the HSCs. In contrast to the ambiguous role of HSCs in bioengineered liver tissues (BLTs), we saw that MSCs played a relatively clear role in the co-cultures by enhancing organoid formation and growth in EM, promoting spontaneous tissue formation, and supporting hepatic function. Although the exact function HSCs performed in BLTs was not clear, we continued this study with the tri-culture (OSM) condition to mimic the cellular complexity and multicellular cooperation of the *in vivo* situation [11,38]. For future studies, it would be interesting to also include Kupffer cells and liver sinusoidal endothelial cells to better mimic the immunological functions of the native liver [39]. Besides mesenchymal cells, Kupffer cells and endothelial cells make up most (∼70%) of the non-parenchymal cells in the liver, and as resident antigen-presenting cells, they are crucial in maintaining tolerance under noninflammatory conditions [40,41].

Besides cellular factors, we showed that the culture method played a vital role in tissue formation. After around 7 days of static culture (SC), we observed that some cells had migrated to the bottom of Matrigel droplets, where they attached and proliferated on the plastic wells, which may have contributed to a higher expression of proliferation markers in the co-cultures during differentiation. To avoid this, we utilized low-attachment plates and placed them on a horizontal shaker as a dynamic suspension culture (DS) method. Remarkably, the DS method not only avoided cell attachment but also promoted spontaneous tissue formation and even enhanced the maturation of BLTs, including the ECM markers *FN* and *VTN* (Fig. 3C and D). The higher *FN* expression in co-cultures probably indicates the potential ECM remodeling by mesenchymal cells, and the trend of upregulated expression of *VTN* in DS is consistent with other hepatocyte markers. This enhanced maturation may be mainly induced by fluidic shear forces caused by the DS condition. Previously, we reported that suspension culture of liver organoids in spinner flasks induced better differentiation compared to SC [10], and Takahashi et al. developed a protocol for the differentiation and maturation of human intestinal organoids completely in suspension [30]. All those studies in suspension underline the importance of a dynamic (micro-) environment including shear stress for tissue formation and regeneration. Our transcriptomic results suggest that the enhanced maturation was not only induced by mechanotransduction changes in the DS condition, but also influenced by “cell-cell and cell-ECM interaction” and “autocrine and paracrine effects” (Fig. S7). Although the autocrine and paracrine effects in OSM seem to be partially suppressed by DS condition, it is not contradictory to strengthened cell-cell interactions. Instead, the DS condition may have enhanced the transport of those autocrine and paracrine molecules, resulting in a negative-feedback loop and lower expression of those signals. In future tissue engineering approaches, continuous fluid flow by perfusion might induce even better tissue fusion and maturation than the DS system used in this study.

Using hydrogels as ECM mimicry not only provides cells with a 3D microenvironment, it can also offer the possibility to adjust mechanical properties and biological functions according to the needs of a particular tissue [42,43]. Although the DS method upregulated the expression of hepatocyte markers on the mRNA level, the key metabolic protein CYP3A4 was hardly detected in Matrigel cultures (Fig. 3E). Then, we replaced Matrigel with PIC-Collagen-I as a well-defined hydrogel. Previously, PIC hydrogels were used for the culture of human liver organoids and mammary gland organoids [19,20] and for the differentiation of cholangiocyte organoids [44]. Collagen-I has been widely applied as a biomaterial in TERM [45], and primary mouse hepatocytes and human HepaRG cells secreted higher levels of urea and albumin in collagen-I microspheres than in most other tested ECM components [26]. In our study, the co-cultures benefited from the combination of the synthetic thermoreversible PIC supplemented with collagen I (PC). PC enabled organoid co-cultures with MSCs and HSCs to spontaneously form compact liver tissues (Fig. 4B) and significantly induced hepatic marker expression, including the expression of CYP3A4 on the protein level (Fig. 4C, E, 7E, Fig. S3A–B). Our results suggest that PC could have promoted BLT maturation via enhanced mechanotransduction (Fig. 7C–D, Fig. 8). Although the expression of some hepatic markers showed a downregulation during extended culture, this is more likely due to the continuous utilization of the DM that is developed for liver organoid differentiation but not ideal for the maintenance of hepatic functions. Importantly, BLTs formed in PC retained a vital portion of hepatic functional markers during extended maintenance of BLTs (Fig. S9E, Fig. 8B). Moreover, PC could have created a stem cell niche with abundant laminin-entactin complex via ECM remodeling and cell-cell and cell-ECM interactions, which may contribute to the extended maintenance of hepatic functions (Fig. S9C–D). Furthermore, since both PIC and collagen-I are thermoresponsive, the hydrogels can easily be removed after liver tissue formation, resulting in xeno-free BLTs suitable for *in vivo* applications.

Recently, two bioprinting- or organ-on-chip-based liver models have been reported to exhibit enhanced hepatic functions compared to their respective controls [46,47]. In contrast to these cancer cell line-based models using Matrigel-containing hydrogels, the BLTs are formed in well-defined hydrogels and more closely recapitulate the cellular complexity of native liver tissue. This is particularly important for studies involving epithelial-mesenchymal crosstalk (*e.g.* related to fibrosis or matrix-remodeling in homeostasis, disease and regeneration) and for future clinical applications. In contrast, the purely epithelial organoid model might be advantageous for studies in which the cellular complexity is less relevant and the main focus is on high functional levels of hepatocyte-like cells, such as drug toxicity studies. A recent study described human liver assembloids generated from organoids and mesenchymal cells [48], which have demonstrated suitability for disease modeling and hold promise for investigating human liver pathophysiology, drug development, early diagnosis and advancing personalized medicine—applications that are likewise envisioned for the BLTs. Notably, the BLTs developed in the present study place particular emphasis on the integration of key bioengineering factors, thereby extending and complementing existing platforms.

Limitations and perspectives: The exact state of the HSCs in BLTs was not fully discovered in this study and it is important to further investigate the activation state of HSCs and their apparently ambiguous role during expansion and differentiation by single cell RNA sequencing. Moreover, the hepatic functional characteristics of the OSM formed BLTs decreased from D8 to D15, along with an increase in mesenchymal markers, which needs to be improved in future. For instance, by optimizing the medium for long-term functional maintenance or by adjusting the cellular composition for a better maintained balance of different cell types. As briefly mentioned above, Kupffer cells and endothelial cells make up ∼70% of the non-parenchymal cells in the liver but are not included in this study. For the future, it is essential to include these non-parenchymal cells, in particular if clinical applications are envisioned, to ensure vascularization and immunological function. Before clinical application is possible, it is also essential to replace the animal-derived collagen used in this study by human recombinant proteins and in an ideal case, to establish the BLTs from autologous donor-derived cells.

In conclusion, we established a straightforward strategy to bioengineer human liver tissues *in vitro* by co-culturing human liver epithelial organoids (ICOs) with mesenchymal cells (HSCs and MSCs) within a well-defined thermoreversible PC hydrogel under dynamic suspension culture on an orbital shaker. This study systematically integrated multiple key bioengineering parameters to examine their combined effects on tissue engineering, using the liver as a showcase: A. Multicellular self-organization under co-culture conditions. B. Dynamic mechanical stimuli generated by orbital shaking. C. Well-defined hydrogels as ECM mimicry. D. Extended maintenance of hepatic functions. The resulting BLTs constitute a valuable platform for toxicity testing, drug screening, and disease modelling, and lay the foundation for bioengineering larger tissues with potential for *in vivo* transplantations. Importantly, the tissue engineering strategy presented here provides a versatile framework for the development of additional biomimetic models not only for the liver but also for other tissue types, and enables systematic investigation of the interplay among key bioengineering factors.

## 4 Materials and methods

### 4.1 Experimental design

**Fig. 9:**
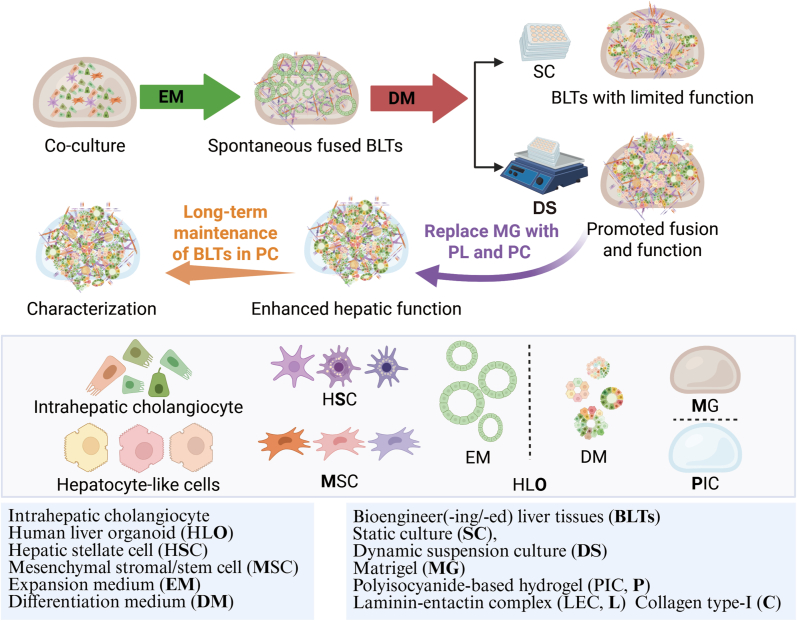
The schematic research line of experimental design for BLTs.

The experimental design of this study is shown in Fig. 9.

### 4.2 Medium preparation

Advanced DMEM/F12 (Gibco) supplemented with 1% (v/v) penicillin-streptomycin (Gibco), 1% (v/v) GlutaMax (Gibco), 10 mM HEPES (Gibco), was used as the basal medium to make organoid initiation medium, expansion medium (EM), differentiation medium (DM), and to wash organoids during passaging or sample collection.

Other major materials used for organoid isolation and maintenance are as follows: type II collagenase (Gibco), dispase (Gibco), Matrigel™ (Corning), and non-attaching 24-well plates (M9312, Greiner, Merck). Certified Fetal Bovine Serum (FBS) was ordered from Gibco (16000-044). Organoid initiation medium was prepared by using 70% EM and adding 30% Wnt-conditioned medium (homemade).

EM was made based on basal medium, supplemented with 2% (v/v) B27 supplement without vitamin A (Invitrogen), 1% (v/v) N2 supplement (Invitrogen), 10 mM nicotinamide (Sigma-Aldrich), 1.25 mM N-acetylcysteine (Sigma-Aldrich), 10% (v/v) R-spondin-1 conditioned medium (the Rspo1-Fc-expressing cell line was a kind gift from Calvin J. Kuo), 10 μM forskolin (FSK, Sigma-Aldrich), 5 μM A83-01 (transforming growth factor b inhibitor; Tocris Bioscience), 50 ng/mL EGF (Invitrogen), 25 ng/mL HGF (Peprotech), 0.1 μg/mL FGF10 (Peprotech) and 10 nM recombinant human (Leu15)-gastrin I (Sigma-Aldrich).

DM was also based on basal medium, supplemented with 1.25 mM N-acetylcysteine (Sigma-Aldrich), 2% (v/v) B27 supplement without vitamin A (Invitrogen), 1% N2 supplement (Invitrogen), 50 ng/mL EGF (Invitrogen), 10 nM recombinant human (Leu15)-gastrin I (Sigma-Aldrich), 25 ng/mL HGF (Peprotech), 100 ng/mL FGF19 (Peprotech), 500 nM A83-01 (Tocris Bioscience), 10 μM DAPT (Selleckchem), 25 ng/mL BMP-7 (Peprotech), and 30 μM dexamethasone (Sigma-Aldrich). DM was changed every 2–3 days for around 8 days.

Hepatic stellate cell growth medium (HSCGM) was made from Dulbecco's modified Eagle's medium (DMEM; Thermo Fisher Scientific), 10% (v/v) FBS (Gibco), and 100 U penicillin/streptomycin (Gibco).

Mesenchymal stromal/stem cell growth medium (MSCGM) was based on DMEM, low glucose, pyruvate (Gibco), supplemented with 10% (v/v) FBS (Gibco), and 100 U penicillin/streptomycin (Gibco).

### 4.3 Organoid isolation

Human liver organoids used in this study were intrahepatic cholangiocyte organoids (ICOs) [49]. Human liver tissues were obtained during liver transplantation at the Erasmus Medical Center Rotterdam, in accordance with the ethical standards of the Helsinki Declaration of 1975. Using the tissue for research purposes was approved by the Medical Ethical Council of the Erasmus Medical Center and informed consent was provided by patients (MEC-2014-060). ICO lines were established and cultured as previously described [8]. Briefly, to establish organoid lines, liver tissues were cut into small pieces, a small part was used for RNA isolation and qPCR assays and the rest followed by enzymatic digestion with 0.125 mg/mL type II collagenase and 0.125 mg/mL dispase in basal medium containing 1% (v/v) FBS. The supernatant was collected every hour. The procedure of tissue digestion and supernatant collection was repeated three times. Collected cells were washed in basal medium containing 1% FBS and centrifuged at 400 x *g* for 5 min. The cells were resuspended in Matrigel at a concentration of ∼500 cells per μL, then seeded as droplets in non-attaching 24-well plates. Organoid initiation medium was added after approximately 15 min incubation at 37°C, 5% CO_2_ in air.

### 4.4 Cell passaging

For organoid expansion, fragments or single cells from organoids were plated within Matrigel droplets, and EM medium was added after gelation. The medium was changed every 2–3 days. Organoids were passaged by mechanical disruption once a week or by single cell dissociation every 10 days, both at an average split rate of 1:3-6 depending on density. In detail, when liver organoids were almost confluent in Matrigel droplets, they were either passaged as fragments or single cells. For passaging as fragments, organoids were collected in 15 mL Eppendorf tubes containing cold basal medium. Then, the organoids were centrifuged at 4°C at 400 x g for 5 min. After removing the supernatant, around 200 μL of cold basal medium was added to the tube and organoids were disrupted mechanically by pipetting up and down until they were small clusters. For passaging with single cells the medium was removed, and 1 mL of Tryple-Express was added to one well of a 12-well plate; then, the Matrigel droplets were disrupted mechanically by pipetting up and down for 7-10 times with 1 mL Eppendorf tips, and the plate was incubated at 37°C for around 30-60 min until the organoids became almost single cells. After that, the single cells or fragments were collected in 15 mL tubes, and the tubes were filled with cold basal medium (for enzymatic passaging with Tryple-Express, the basal medium contained 3-5% (v/v) FBS) and centrifuged again to get a pellet of organoid cells. Once the supernatant was removed, the organoid cells were resuspended in Matrigel and plated in a 24-well plate (50 μL/well). EM was added (500 μL/well) after approximately 15 min incubation at 37°C. All cultures were kept in a humidified atmosphere of 95% air and 5% CO_2_ at 37°C.

For HSCs and MSCs passaging, a 1:4–1:8 split ratio was performed once every 5–10 days. More specifically, once the cultures were almost confluent, cells were surpassed by using Tryple-Express (Gibco) for 5 min at 37°C. Cells were collected and centrifuged at 400 x *g* for 5 min at 4°C. After centrifugation, the supernatants were removed, and the cells were cultured in T75 flasks with 10 mL of HSCGM or MSCGM for HSCs and MSCs, respectively. All cultures were kept in a humidified atmosphere of 95% air and 5% CO_2_ at 37°C.

### 4.5 Cell seeding and hydrogel preparation

Matrigel (Corning®, 356231), PIC (1k-PIC-P, NCN01, Noviogel), and Laminin-entactin complex (LEC, Corning) were thawed at 4°C 1-2 h before use. The preparation of single cells from organoids, HSCs, and MSCs was described above. Once the single cells were ready for use after cell counting, the required amounts of cell suspensions were added to 1.5 mL Eppendorf tubes. Then, the cell suspensions were centrifuged at 400 x *g* for 5 min at 4°C to get cell pellets for later use. During the waiting steps, the Type I Collagen (collagen-1, Sigma-Aldrich, 08-115) was diluted to a certain concentration so that a fixed volume could be added to each mixture of cells and hydrogels. The final concentrations of Matrigel, PIC, LEC, and collagen-1 were as follows: Matrigel (MG, 10 mg/mL), PIC (1 mg/mL), LEC (3 mg/mL), and collagen-1 (1.5 mg/mL). The cell concentrations of each type of cell in different hydrogels were kept consistent: 1200 organoid cells/μL, 200 HSCs/μL, and 200 MSCs/μL, which means the ratio of different types of cells was: Organoid: HSC: MSC = 1200: 200: 200 or 6: 1: 1. There were in total six different combinations of cultures: organoid only (O), organoid with HSCs (OS), organoid with MSCs (OM), organoid with HSCs and MSCs (OSM), HSCs only (S), and MSCs only (M).

After mixing the cells with hydrogel solutions on ice, the mixtures were seeded in droplets in prewarmed 24-well plates (4-7 droplets/50 μL/well). Plates were incubated at 37°C for around 15 min for the gels to solidify. Once the hydrogels became solid, EM was added (500 μL/well). Then, EM was refreshed every 2-3 days for 10-11 days. All cultures were kept in a humidified atmosphere of 95% air and 5% CO_2_ at 37°C.

### 4.6 Hydrogel characterization

Rheological measurements were conducted as described previously [19]. The discovery HR-2 rheometer (TA instruments) was utilized to test the mechanical properties of Matrigel, PIC, and PIC + Collagen I (PC) hydrogels. A 20 mm parallel plate (aluminium-105381) was used for all temperature-controlled measurements. A geometry diameter of 20 mm and a measuring gap of 300 μm were applied. For hydrogel gelation measurements, 150 μL of each hydrogel premix was added onto the plate and conditioned for 2 min at 4°C. Then, the plate was heated (strain 1%, angular frequency 1.0 rad s^−1^) to 37°C at a speed of 7°C min^−1^, and then continuously measured for another 10 min at 37°C. For thermoreversible characterization, hydrogel gelation was carried out as described above. After maintaining at 37°C for 10 min, the plate was cooled down to 4°C at a rate of 7°C min^−1^. Once the temperature of the plate reached 4°C, it was held for another 10 min to characterize if the samples could flow again. Hydrogel average mesh size (ξ) was calculated using the elastic blob theory: $\xi ={\left(\frac{G^{\prime }N_{av}}{RT}\right)}^{-1∕3}$ with N_av_ being Avogadro's constant, R the molar gas constant, and T the absolute temperature in K.

### 4.7 Differentiation

To induce hepatic functions, liver organoids and their co-cultures were primed for 3 days in EM with the addition of 25 ng/mL BMP-7 (Gibco), after which the medium was changed to DM. DM was changed every 2–3 days for 8 days or 14 days (for extended maintenance assays).

### 4.8 Static culture and dynamic suspension culture

For expansion, both the static culture (SC) and dynamic suspension culture (DS) conditions were cultured in a standard culture stove in respectively standard and low-attachment plates (Greiner). After expansion, the DS condition was placed on a horizontal shaker at a speed of 70 rpm for differentiation. Both conditions were kept in a humidified atmosphere of 95% air and 5% CO_2_ at 37°C.

### 4.9 RNA isolation, cDNA synthesis, and qPCR assays

Trizol Reagent (Ambion, by Life Technologies, 15596018) was used to isolate RNA from organoids and other samples following the manufacturer's instructions. Firstly, 0.5 mL of Trizol was added to each well (24-well plate) of organoids. After pipetting up and down for a couple of times, the cell suspensions were transferred into 1.5 mL RNase-free Eppendorf tubes. RNA quality and quantity were measured with DS-11 Spectrophotometer (DeNovix). Complementary DNA (cDNA) was synthesized with the iScript™ cDNA synthesis kit (Bio-Rad, 1708891) following the manufacturer's instructions. Quantitative real-time PCR (qPCR) was used to determine relative expression of target genes using validated primers (Table 1) using the SYBR Green method (iQ SYBR Green Supermix, Bio-Rad). Normalization was carried out using reference genes *GAPDH* and ribosomal protein L19 (*RPL19*).

**Table 1:** List of primers for gene expression profiling.

Target	Forward primer	Reverse primer	Annealing Temp [°C]	Product size [bp]
*GAPDH*	CAAGATCATCAGCAATGCCT	CAGGGATGATGTTCTGGAGAG	60	194
*RPL19*	ATGAGTATGCTCAGGCTTCAG	GATCAGCCCATCTTTGATGAG	64	150
*ALB*	GTTCGTTACACCAAGAAAGTACC	GACCACGGATAGATAGTCTTCTG	64	144
*BSEP*	TTGAGACAATAGACAGGAAACC	TCTGGAAGGATAATGGAAGGT	60	116
*CYP3A4*	CACAGGCTGTTGACCATCAT	TTTTGTCCTATAAGGGCTTT	60	92
*HNF4a*	CATGTACTCCTGCAGATTTAGCC	CTTCCTTCTTCATGCCAGCC	60	110
*MDR1*	AATGATGCTGCTCAAGTTAAAGGG	TCAGTAGCGATCTTCCCAGAACC	60	239
*MRP2*	GCCAACTTGTGGCTGTGATAGG	ATCCAGGACTGCTGTGGGACAT	60	139
*FN1*	CCCATCAGCAGGAACACCTT	GGCTCACTGCAAAGACTTTGAA	60	82
*VTN*	TGACCAAGAGTCATGCAAGGG	ACTCAGCCGTATAGTCTGTGC	60	116
*LGR5*	GCAGTGTTCACCTTCCC	GGTCCACACTCCAATTCTG	64	82
*Ki67*	GCTACTCCAAAGAAGCCTGTG	AAGTTGTTGAGCACTCTGTAGG	60	143
*ECAD*	AGGCCAAGCAGCAGTACATT	ATTCACATCCAGCACATCCA	60	110
*KRT19*	CTTCCGAACCAAGTTTGAGAC	AGCGTACTGATTTCCTCCTC	64	183
*YAP*	TAGCCCTGCGTAGCCAGTTA	TCATGCTTAGTCCACTGTCTGT	60	177

### 4.10 Transcriptomic analysis

For whole transcriptome sequencing, RNA was isolated from organoids and other samples using the Trizol Reagent according to the manufacturer's instructions. A minimum of 60 μL of RNA with RNA Integrity Numbers (RIN) ≥ 7, at a concentration of at least 10 ng/μL, was submitted for mRNA library preparation using poly(A) enrichment and sequenced at the Utrecht Sequencing Facility, following their internal protocols. Libraries were sequenced on the Illumina NextSeq2000 platform, generating paired-end reads (2 × 50 bp) with approximately 20 million reads per sample and 1 billion reads per flowcell. The mRNA reads were aligned to the human reference genome using STAR (version 2.4.2a, https://github.com/UMCUGenetics/RNASeq-NF/). Datasets were normalized for library size using the trimmed mean of M values (TMM). Fold changes were calculated with edgeR [50], and gene expression comparisons employed the exact test to compute p-values, adjusted for a 5% false discovery rate (FDR) using the Benjamini-Hochberg method. Donor origin was included as a covariate in the design matrix to correct for inter-individual variability. Genes with adjusted p-values <0.05 were deemed differentially expressed. Principal component analysis (PCA) plots and heatmaps of differentially expressed genes were generated using ggplot2 [51] (https://cran.r-project.org/web/packages/ggplot2/citation.html. Click or tap if you trust this link.">https://cran.r-project.org/web/packages/ggplot2/citation.html]). Volcano plots were produced with EnhancedVulcano [52]. (EnhancedVolcano: Publication-ready volcano plots with enhanced colouring and labeling. R package version 1.24.0, https://github.com/kevinblighe/EnhancedVolcano. Click or tap if you trust this link.">https://github.com/kevinblighe/EnhancedVolcano.), applying thresholds of P = 0.0001 and fold change (FC) = 4. Gene Set Enrichment Analysis (GSEA) [53] was conducted to identify enriched Gene Ontology (GO) terms among the differentially expressed genes. The gene interaction network and GO analysis for the top 200 most differentially expressed gene analyses were conducted via ToppGene https://toppgene.cchmc.org/enrichment.jsp. To estimate cell type composition, a curated set of marker genes specific to major liver cell types was used. These included Hepatocytes (*ALB, HNF4A, CYP3A4, ABCC2, CPS1*), Epithelial Cells (*KRT19, SOX9, NCAM1, ECAD, EPCAM*), Stem/Progenitor & proliferative Cells (*PROM1, LGR5, AFP, SOX9, ITGA6, MKI67*), MSCs (*NT5E, THY1, ENG, MCAM, DCN, VIM*), and HSCs (*LRAT, GFAP, MCAM, ACTA2, COL1A1, DES, PDGFRB, RBP1*). Marker genes were selected based on published literature and publicly available single-cell RNA sequencing datasets.

### 4.11 Immunofluorescent and histological staining

For immunofluorescent (IF), H&E and Periodic acid-Schiff (PAS) staining, organoids and bioengineered liver tissues (BLTs) were fixed with 4% (v/v) neutral buffered formalin containing 0.1% eosin and incubated at 37°C overnight. Fixed samples were dehydrated and embedded in paraffin or stored in 70% (v/v) ethanol at 4°C for up to 1 month; 4 μm thick paraffin sections were prepared for IF, H&E, and PAS staining. PAS staining and H&E staining were conducted as previously described [10] to evaluate the function of BLTs for glycogen storage and to compare the histology, respectively. To start the IF staining procedure, the paraffin sections were first heated at 62°C for 10 min and dewaxed by xylene, followed by rehydration in gradient ethanol concentrations from 100% to 70%. Then, sample sections were incubated in antigen retrieval solution for 30 min at 98°C. After balancing to room temperature, sample sections were treated with NH_4_Cl solution (20 mM) for 10 min to reduce background autofluorescence and blocked with 10% (v/v) goat serum for 1 h to avoid non-specific antibody binding. Next, primary antibodies against Albumin (ALB), Cytochrome P450 Family 3 Subfamily A Member 4 (CYP3A4), E-cadherin (ECAD), and Keratin 19 (K19) were added to the sections and incubated overnight at 4°C. After being washed with PBS containing 0.1% (v/v) Tween 20 for three times, sample sections were incubated with secondary antibodies (5 μM), including mouse anti-rabbit Alexa Fluor® 488, mouse anti-rabbit Alexa Fluor647, rabbit anti-mouse Alexa Fluor488 and rabbit anti-mouse Alexa Fluor647 (all from Molecular Probes). Nuclei were stained with DAPI (0.5 μg/mL, Sigma Aldrich). Detailed information on applied antigen retrieval methods, antibodies, dilutions, and incubation times are listed in Table 2.

**Table 2:** List of antibodies used.

Primary antibodies diluted in antibody diluent (Dako) for IF					
Antigen	Source and cat. number	Raised in	Dilution	Antigen retrieval	Incubation
ALB	Sigma, A6684	Mouse	1:1000	TE buffer for 30 min at 98°C	O/N at 4°C
CYP3A4	Abcam, ab124921	Rabbit	1:100		
E-cadherin	BD Bioscience, 610181	Mouse	1:100		
KRT19	Abcam, ab76539	Rabbit	1:150		
BSEP	Invitrogen, PA5-78690	Rabbit	1:500		
KRT18	Santa Cruz, sc-51582	Mouse	1:100		
MRP3	Abcam, ab3375	Mouse	1:100		
MDR1	Novus bio, NBP1-90291	Rabbit	1:200		
MRP2	Cell Sciences, MON9027	Mouse	1:75		
VIM	Abcam, ab8979	Mouse	1:100		
VIL	Abcam, ab201989	Mouse	1:100		
COL3A	BD Bioscience, 610181	Mouse	1:100		
Laminin 1	Abcam, ab11571	Rabbit	1:100		
YAP	Abcam, ab205270	Rabbit	1:100		
**Secondary antibodies diluted in antibody diluent (Dako)**					

**Antigen**	**Source and cat. number**	**Raised in**	**Dilution**	**Incubation**	

Anti-mouse Alexa 488	ThermoFisher A-11029	Goat	1:200	1 h at RT	
Anti-rabbit Alexa 488	ThermoFisher A-11034				
Anti-mouse Alexa 568	ThermoFisher A-11004				
Anti-rabbit Alexa 568	ThermoFisher A-11036				

### 4.12 TEM analysis

Organoid-only (O-) and OSM-derived BLTs in Matrigel and PIC + collagen I (PC) hydrogels were fixed in half-strength Karnovsky fixative (2.5% Glutaraldehyde (EMS) + 2% Formaldehyde (Sigma)) in pH 7.4 0.1M PHEM buffer at RT for 2 h. Fixed samples were rinsed and stored in 1% FA at 4°C until further processing. Post-fixation was performed with 1% OsO4, 1.5% K3Fe(III)(CN)6 in 1 M phosphate buffer pH 7.4 for 2 h. BLTs were then dehydrated in a series of acetone (70% overnight, 90% 15 min, 96% 15 min, 100% 3 × 30 min), and embedded in Epon (SERVA). 70 nm ultrathin sections were cut (Leica Ultracut UCT), collected on formvar and carbon-coated transmission electron microscopy (TEM) grids, and stained with uranyl acetate and lead citrate (Leica AC20). Micrographs were collected on a JEM1011 (JEOL) equipped with a Veleta 2 k × 2 k CCD camera (EMSIS, Munster, Germany) or on a Tecnai12 (FEI Thermo Fisher) equipped with a Veleta 2 k × 2 k CCD camera (EMSIS, Munster, Germany) and operating SerialEM software. The alignment and montage of the stitched images were established in the IMOD software (https://bio3d.colorado.edu/imod/. Click or tap if you trust this link.">https://bio3d.colorado.edu/imod/). The images were visualized and analyzed in FiJi [54].

### 4.13 Microscopy and images analysis

Imaging of the cultures was performed using an EVOS FL Cell Imaging System (Life Technologies). Bright-field images were taken to track organoid morphology throughout expansion in different cellular formulations. Images were also taken to compare the morphology of organoids and their co-cultures, in EM and DM, embedded in Matrigel or PIC hydrogels, under SC or dynamic suspension culture (DS) conditions.

IF, H&E and PAS stainings were imaged with an Olympus BX51 microscope in combination with an Olympus DP73 camera.

Image analysis for cell counting and fluorescence intensity: Fluorescence image analysis was performed using the software ImageJ (National Institutes of Health, USA). All images were acquired under identical microscope settings (including exposure time, gain, and intensity) to ensure comparability between groups. All image processing parameters, including threshold values and analysis settings, were kept constant across all samples to ensure consistency. Data were exported from ImageJ and further analyzed using GraphPad Prism software.

### 4.14 Intracellular and medium protein measurement

To quantify the intracellular and medium levels of ALB, ALAT, ASAT, and GLDH, BLTs were differentiated in PC or Matrigel droplets for 8 or 15 days as described above. BLTs were provided with fresh DM 24 h before being lysed in MilliQ water. ALB, ALAT, ASAT, and GLDH were measured in the cell lysates and for ASAT, also the medium supernatant, using a DxC-600 Beckman chemistry analyzer (Beckman Coulter). Values were normalized to live cell numbers.

### 4.15 Ammonium elimination assay

For ammonium elimination assays, both organoid-only (O-) and OSM-derived BLTs were differentiated in PC or Matrigel droplets for 15 days as described above. BLTs were incubated with DM supplemented with NH_4_Cl (2 mm) for 24 h on both D8 and D15. After 24 h, D8 and D15 media samples were harvested and stored at −20°C, respectively. Afterward, Tryple-Express (Gibco) was added to each well, and BLTs were trypsinized for cell counting. Cell counts were carried out using the TC20 automated cell counter (Bio-Rad). Viable cells were determined using the trypan blue exclusion assay. Ammonium concentrations were measured with the urea/ammonia Assay Kit (Megazyme). As a control, DM containing NH_4_Cl (2 mM) was incubated for 24 h without cells. Ammonia elimination rates were normalized to live cell numbers.

### 4.16 Metabolic activity analysis with LC-MS/MS

The drug-metabolizing activity of BLTs was evaluated using a cocktail of parental compounds, as previously described [55]. These included 5 μM Midazolam (BUFA; metabolites: 1′-Hydroxymidazolam, Sigma-Aldrich; and 1′-Hydroxymidazolam glucuronide, LGC Standards), 15 μM Phenacetine (Sigma-Aldrich; metabolite: Acetaminophen, Sigma-Aldrich), and 12 μM 7-Hydroxycoumarin (Umbelliferone, Sigma-Aldrich; metabolite: 7-Hydroxycoumarin glucuronide, LGC Standards). Cultures were exposed to the cocktail in DM in a humidified atmosphere at 37°C and 5% CO2 for 24 h 200 μL culture medium was collected in the 1.5-mL Short Thread Vials with the ND9 Short Thread Screw Caps (BGB Analytik), diluted 8 times with ultrapure MeOH, vortexed and stored at −20°C until the quantification of the parental compounds and metabolites using LC-MS/MS, as previously reported [55]. Briefly, prior to the analysis, samples were centrifuged for 10 min at 1500 g to precipitate any protein. Standards of the parental compounds and metabolites were prepared in the same medium (matrix) as samples. 1 μL solution was injected for measurements. Standards and samples were analyzed in a single run using a Shimadzu triple-quadrupole LCMS 8050 system with two Nexera XR LC-20AD pumps, a Nexera XR SIL-20AC autosampler, a CTO-20AC column oven and an FCV-20AH2 valve unit (Shimadzu). The compounds were separated on a Synergi Polar-RP column (150 × 2.0 mm, 4 μm, 80 Å) with a 4 × 2 mm C18 guard column (4 × 2 mm, Phenomenex). The mobile phase consisted of 0.1% (*v*/*v*) formic acid in ultrapure water (A) and 0.1% (*v*/*v*) formic acid in MeOH (B), and was set as 100% A (0-1 min), 100% to 5% A (1-8 min), 5% A (8-9 min), 5% to 100% A (9-9.5 min) and 100% A (9.5-12.5 min). The total run time was 12.5 min, and the flow rate was 0.2 mL/min. Peaks were integrated using the LabSolutions software (Shimadzu). For each compound, a limit of quantification (LOQ) was determined based on a standard curve and a limit of detection (LOD) was determined based on a noise signal. For each sample set, a dose (time 0 h) and a blank (negative control) were measured. Amounts of metabolites were normalized to live cell numbers.

### 4.17 Statistical analyses

Gene expression and functional analysis results were analyzed in the Excel sheets (Microsoft) and then transformed into graphs using GraphPad Prism 9. Tukey's multiple comparisons test by One-Way or Two-Way ANOVA Multiple comparisons. The p-values (significance set at 0.05) are indicated in the respective figures. All average values described in the text and figures are mean ± SD (standard deviation).

## Funding

This work was supported by 10.13039/501100003246Dutch Research Council 10.13039/501100003246NWO VENI (016.Veni.198.021) (K.S.), 10.13039/501100004543China Scholarship Council (CSC201808310180) (S.Y.), 10.13039/501100004543China Scholarship Council (CSC201808620130) (Z.W.)

## CRediT authorship contribution statement

**Shicheng Ye:** Conceptualization, Data curation, Formal analysis, Funding acquisition, Investigation, Methodology, Project administration, Validation, Visualization, Writing – original draft, Writing – review & editing. **Zhenguo Wang:** Data curation, Formal analysis, Funding acquisition, Investigation, Methodology, Visualization, Writing – review & editing. **Maarten Dirk Delemarre:** Data curation, Investigation, Methodology, Visualization, Writing – review & editing. **Nalan Liv:** Data curation, Formal analysis, Methodology, Visualization, Writing – review & editing. **Antoinette van den Dikkenberg:** Methodology. **Tina Vermonden:** Methodology, Writing – review & editing. **Luc van der Laan:** Resources, Writing – review & editing. **Jos Malda:** Writing – review & editing. **Bart Spee:** Resources, Writing – review & editing. **Frank G. Van Steenbeek:** Data curation, Formal analysis, Methodology, Software, Visualization, Writing – review & editing. **Kerstin Schneeberger-Verjaal:** Funding acquisition, Investigation, Project administration, Resources, Supervision, Writing – review & editing.

## Declaration of competing interest

The authors declare that they have no known competing financial interests or personal relationships that could have appeared to influence the work reported in this paper.

## Data Availability

Data will be made available on request.
