# Supplementary material for: Integrated mesenchymal and extracellular cues drive bioengineered liver tissue formation and function
Source: Mater Today Bio. 2026 Jun 9;39:103328. doi: 10.1016/j.mtbio.2026.103328 (PMC13277544; doi:10.1016/j.mtbio.2026.103328)
Supplement: Multimedia component 1 [file mmc1.docx]

**Supplementary Figures and Tables**


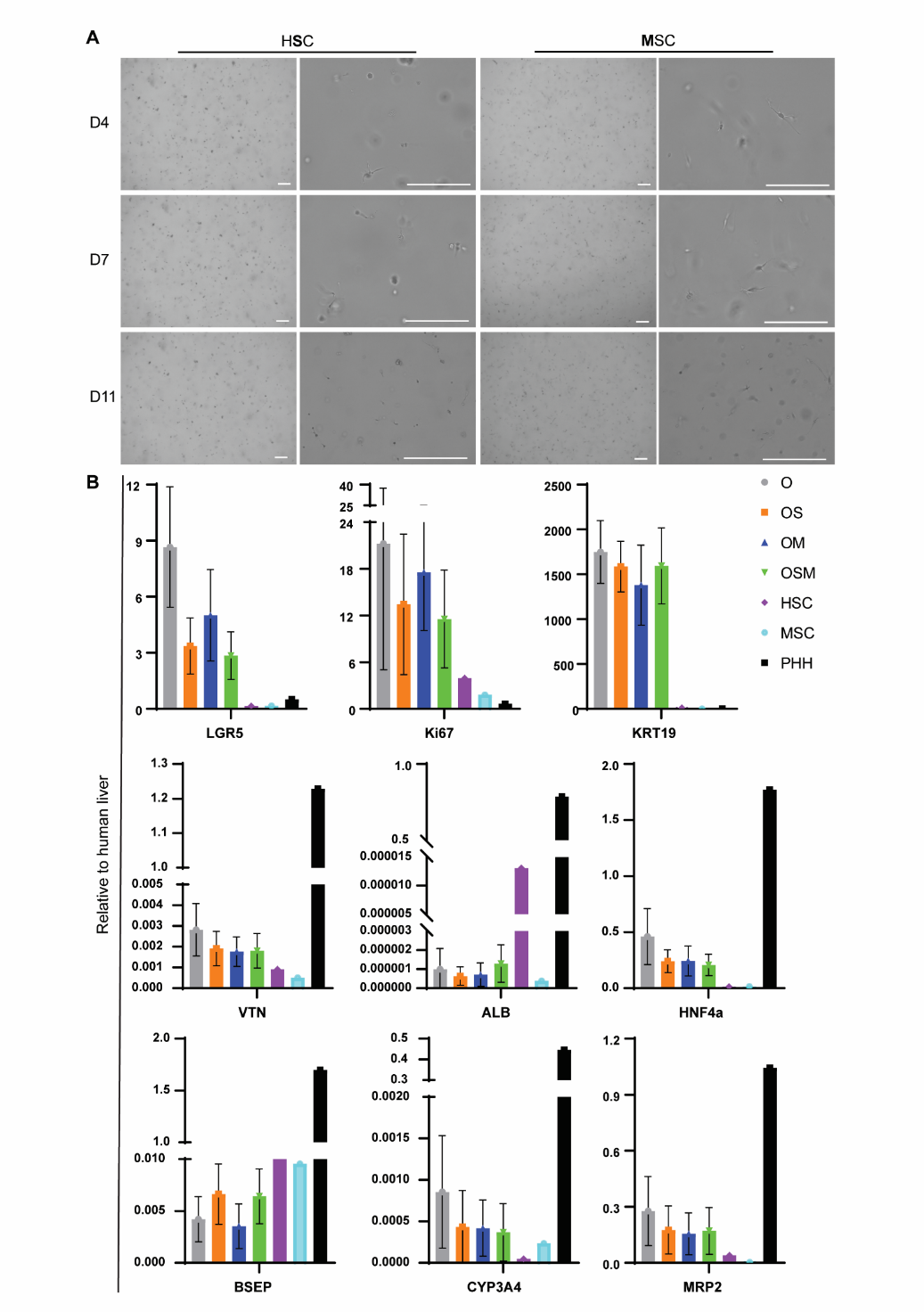


***Fig. S1.*** ***Expansion of mesenchymal cells in Matrigel. n=1.*** *(A) Bright-field pictures showing the morphology of mesenchymal cells (HSC & MSC) in expansion medium (EM) at three time points, D4 (top), D7 (middle) and D11 (bottom). (B) Gene expression of mesenchymal cells only (HSC or MSC) expanded in Matrigel for 11 days, compared to primary human liver tissues. Stem cell/progenitor marker LGR5, proliferative marker Ki67, ductal marker KRT19, and hepatocyte markers HNF4a, ALB, CYP3A4, BSEP, MRP2, and VTN were used for the qPCR assays.*


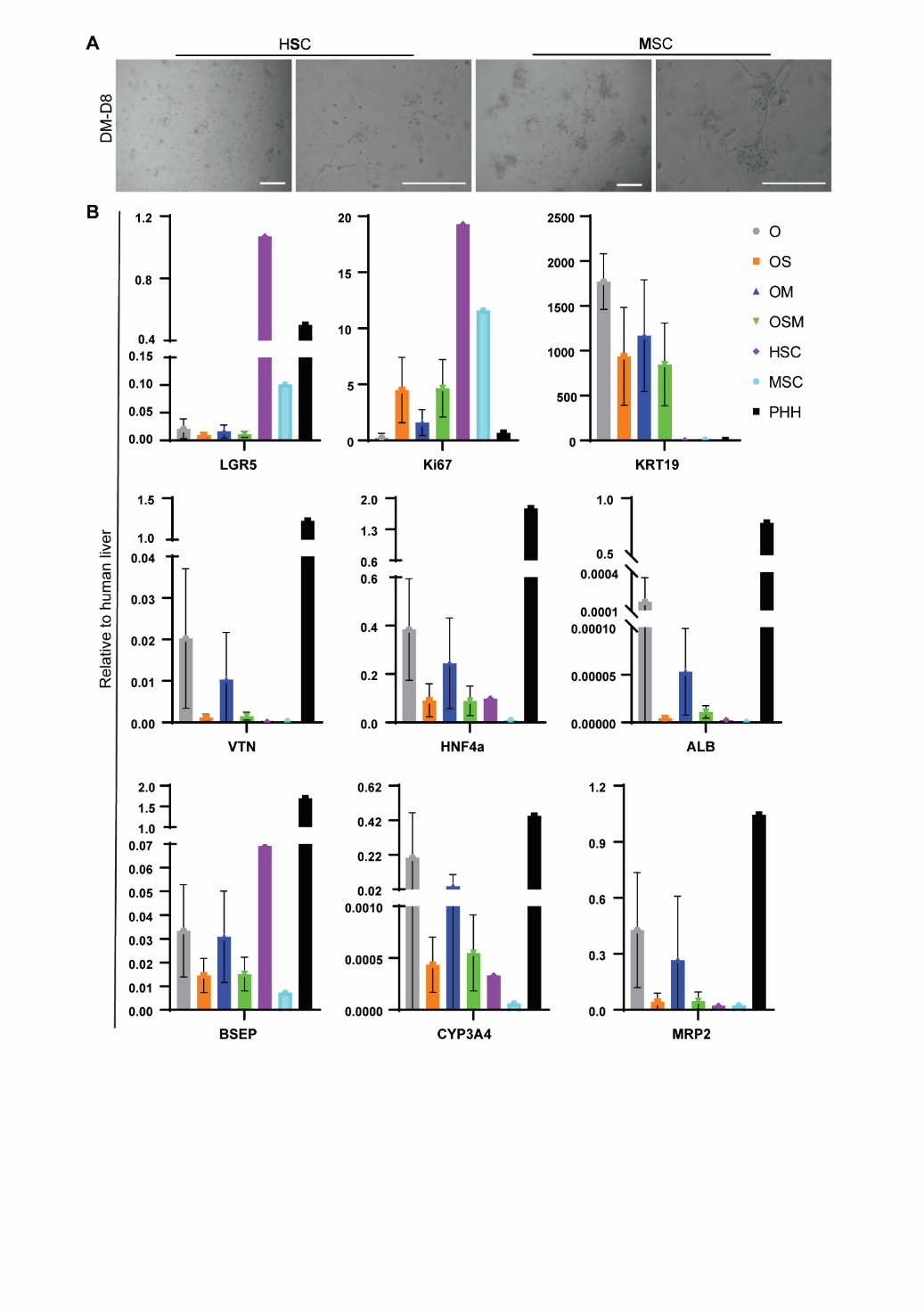


***Fig. S2.*** ***Mesenchymal cells in differentiation medium in Matrigel. n=1.*** *(A) Bright-field pictures showing the morphology of mesenchymal cells (HSC & MSC) in differentiation medium (DM) for 8 days. (B) Gene expression of mesenchymal cells only (HSC or MSC) in Matrigel for 8 days, compared to primary human liver tissues. Stem cell/progenitor marker LGR5, proliferative marker Ki67, ductal marker KRT19, and hepatocyte markers HNF4a, ALB, CYP3A4, BSEP, MRP2, and VTN were used for the qPCR assays.*


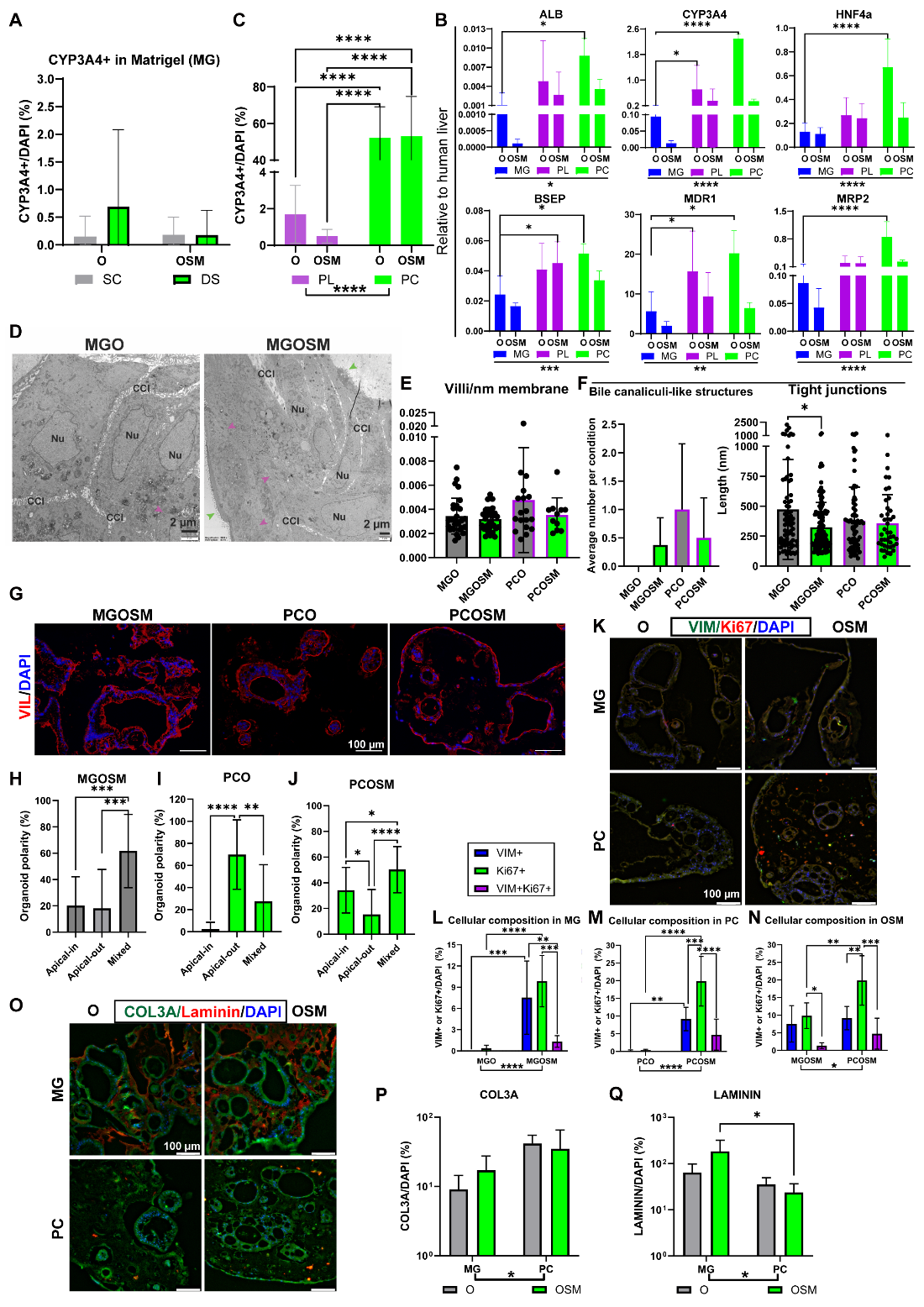


***F******ig. S3. Gene expression in different hydrogels and quantifications of IF and TEM images. (****A) Quantification of CYP3A4+ cells, related to Fig. 3E. (B) Direct comparison of hepatic gene expression in different hydrogels (i.e., MG vs PL vs PC from Fig. 3C and Fig. 4C). (C) Quantification of CYP3A4+ cells, related to Fig. 4E. (D) TEM images of MGO and MGOSM. (E) Quantification of villi density, related to Fig. 5J. (F) Quantification of tight junction length and bile canaliculi-like structures, related to Fig. 5J. (G) IF staining on the apical polarity marker Villin-1 (VIL) for conditions MGOSM, PCO, and PCOSM. (H-J) Quantification of organoid polarity by VIL localization (K) IF staining on the mesenchymal marker Vimentin (VIM) and proliferative marker Ki67. (L-N) Quantification of VIM+, Ki67+, and VIM+Ki67+ double-positive cells. (O) IF staining on major ECM proteins Collagen type III (COL3A) and LAMININ. (P-Q) Quantification of COL3A and Laminin expression levels. n=3-5*


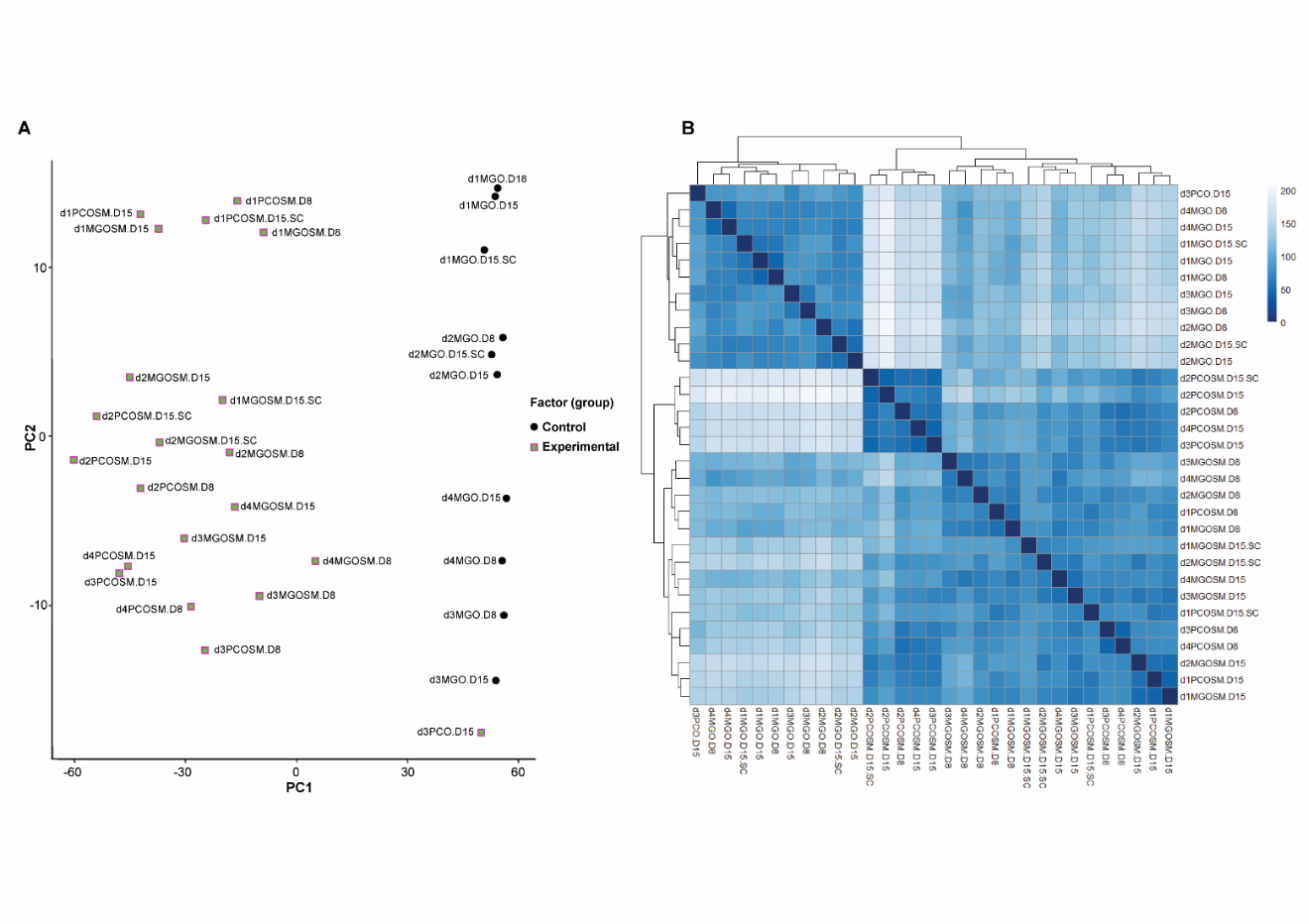


***Fig. S4. Transcriptomic analysis of all samples used for bulk RNA-seq.*** *(A) PCA graph shows the contribution of the top two principal components to the variance in mRNA expression, with differences between the organoid-only (O) and co-culture (OSM) conditions the most obvious. The second obvious differences are between Matrigel (MG) and PIC+Collagen (PC) hydrogels while the differences between static culture (SC) and dynamic suspension (DS) culture are even less pronounced than donor variations. (B) Pearson correlation among all the samples in the data set shows overall clear separation by influencing factors by cellular complexity (O vs OSM), hydrogels (MG vs PC), donors (d1, d2, d3, d4) and less clear separation by culture conditions (SC vs DS) and differentiation time (D8 vs D15). n=4*


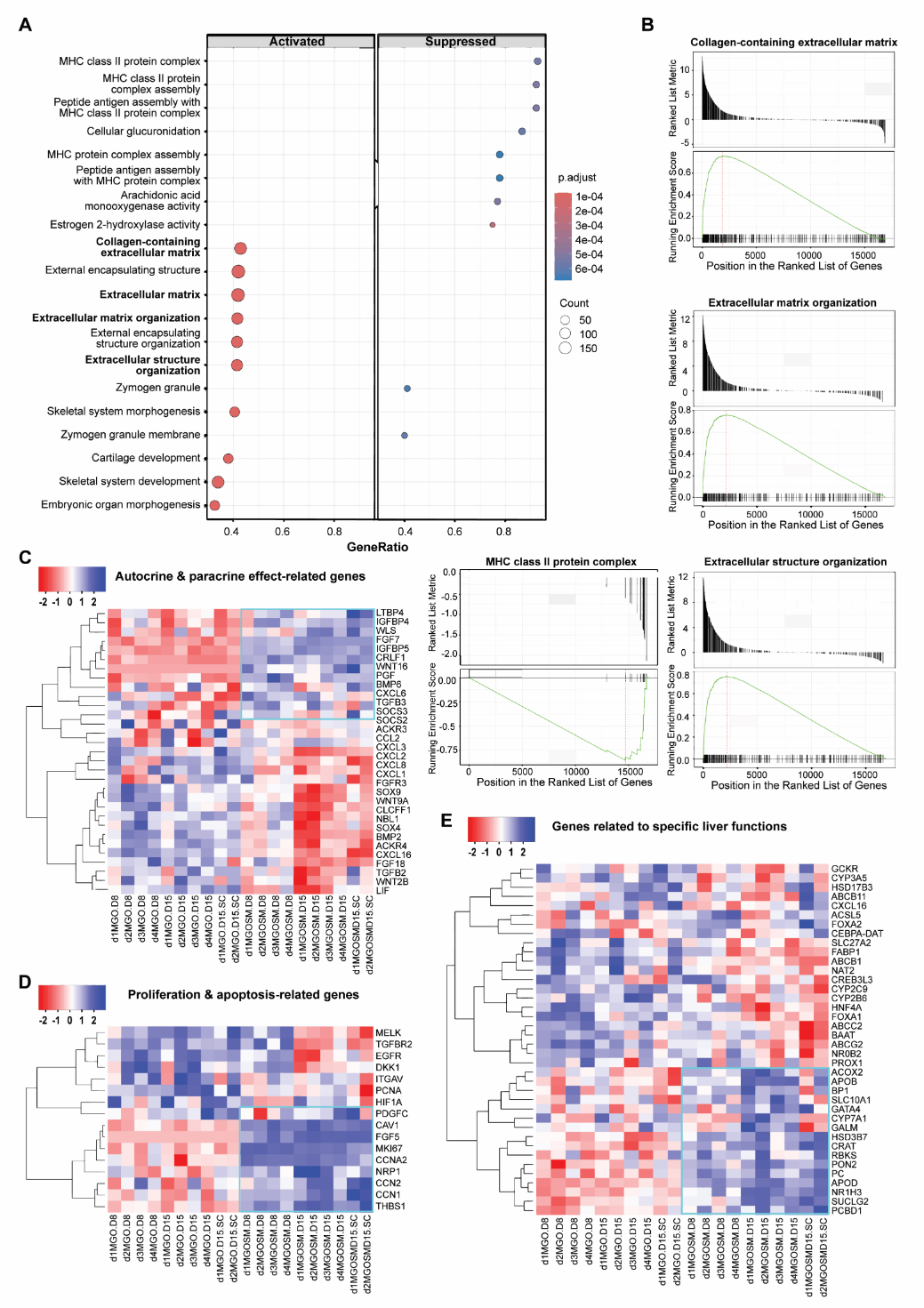


***Fig. S5. Transcriptomics reveal enhanced ECM remodeling by mesenchymal cells****. (A) Gene Ontology (GO) analysis reveals the top involved signaling pathways for the comparison between O and OSM conditions. (B) GSEA plots reveal the potential signaling pathways involved in the functional enhancement of OSM based on the GO gene set. Heatmaps based on the previously reported genes that are associated with autocrine & paracrine effect (C), proliferation & apoptosis (D), or specific liver functions (E). n=4*


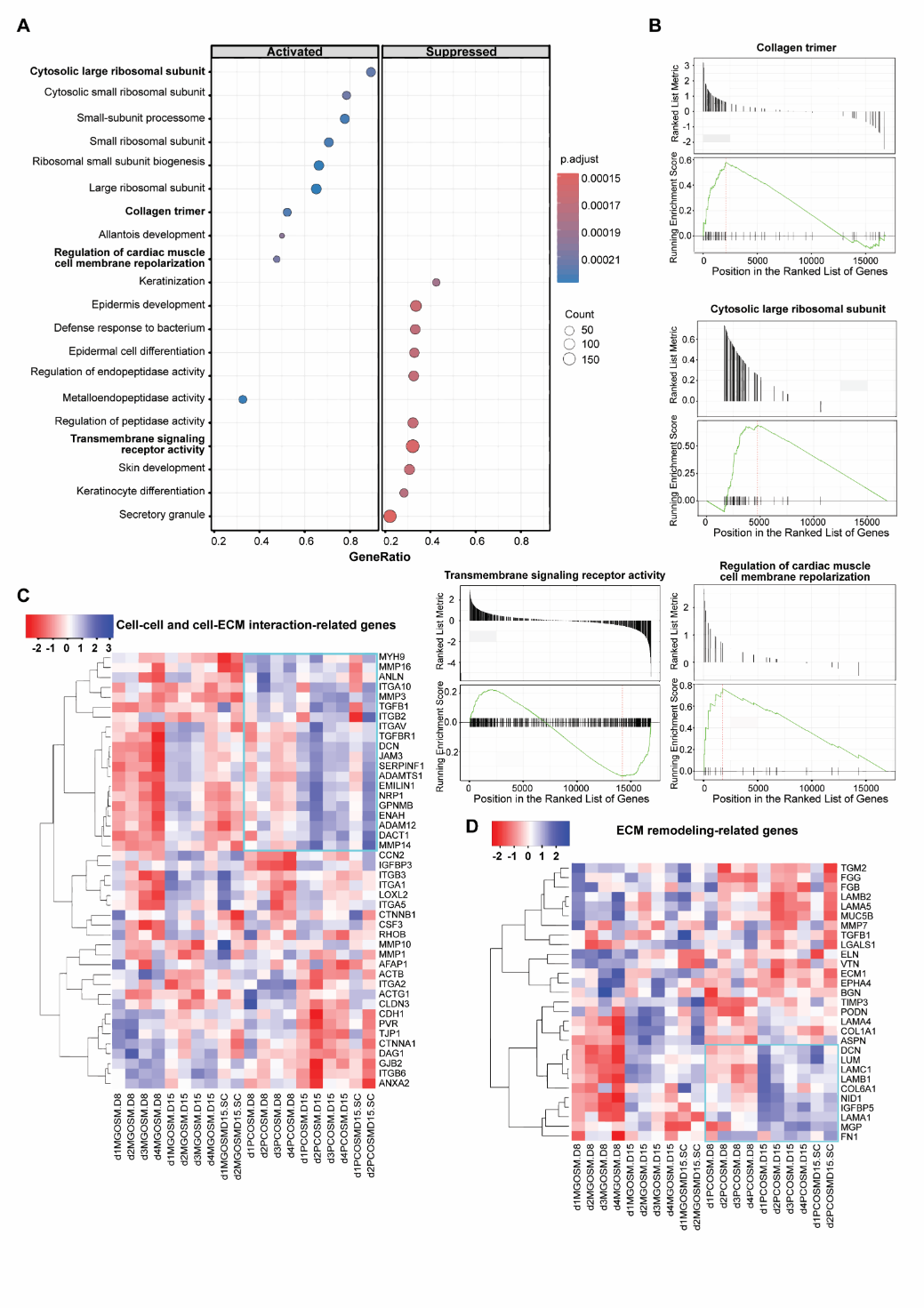
***Fig. S6. Transcriptomic comparison of BLTs in Matrigel and PC****. (A) GO analysis reveals the top involved signaling pathways for all genes comparing MG and PC conditions. (B) GSEA plots reveal the potential signaling pathways involved in the functional enhancement of PC based on the GO gene set. Heatmaps were made out of genes previously reported to be associated with cell-cell & cell-ECM interaction (C) and ECM-remodeling (D). n=4*


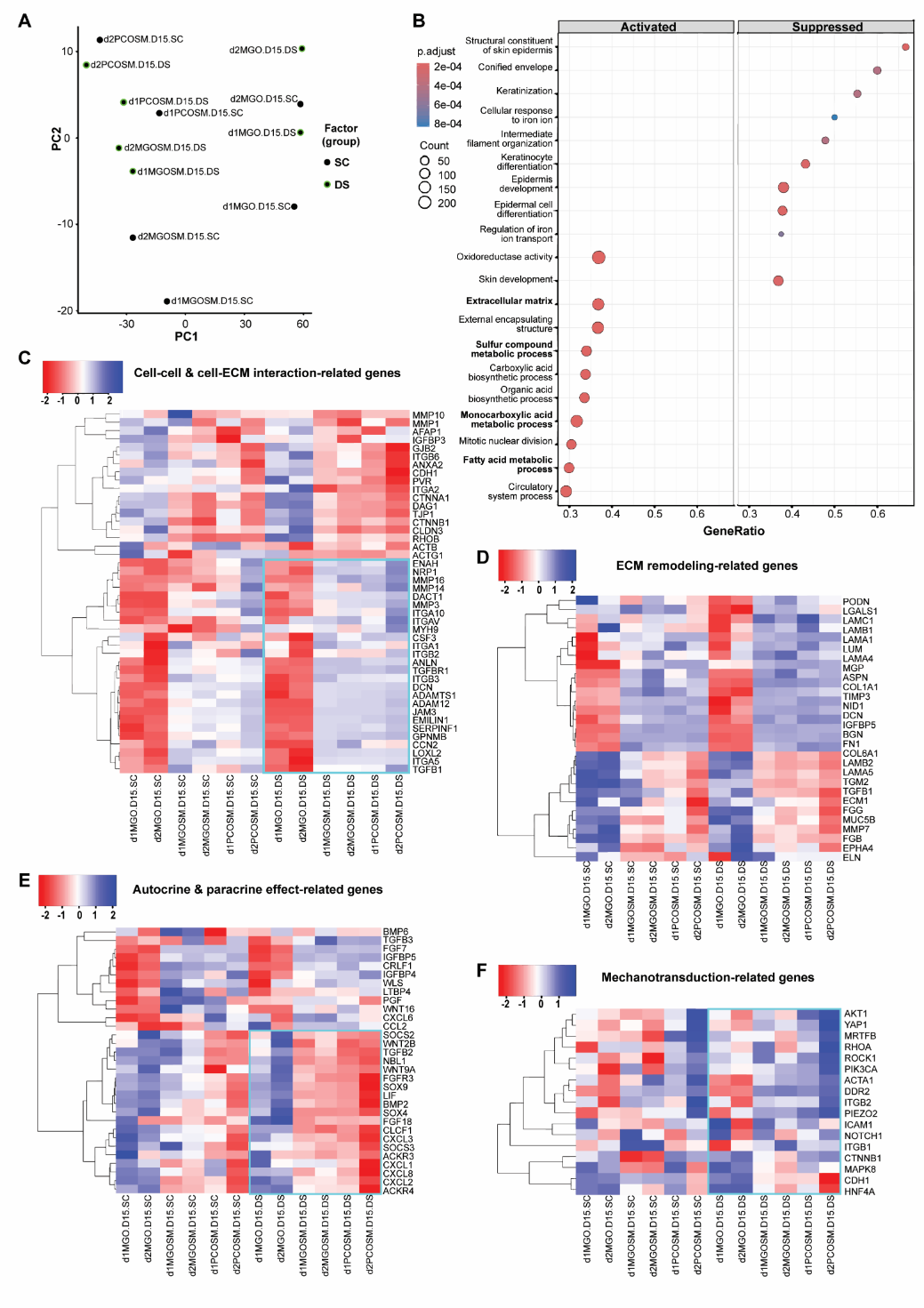


***Fig. S7. Transcriptomics reveal stronger influences on OSM than on O by dynamic suspension culture****. (A) PCA graph shows the contributions of the top two principal components to the variance in mRNA expression between the static culture (SC) and dynamic suspension (DS) conditions with Matrigel (MG) or PIC + Collagen (PC) to form BLTs. The relation between the top two principal components (PC1 and PC2) highlights the separation of samples by culture conditions (SC or DS) and donors (d1 or d2). (B) GO analysis reveals the top involved signaling pathways for all genes comparing SC and DS conditions. Heatmaps were made out of genes previously reported to be associated with cell-cell & cell-ECM interaction (C), ECM-remodeling (D), autocrine & paracrine effect (E), and mechanotransduction (F). n=4*


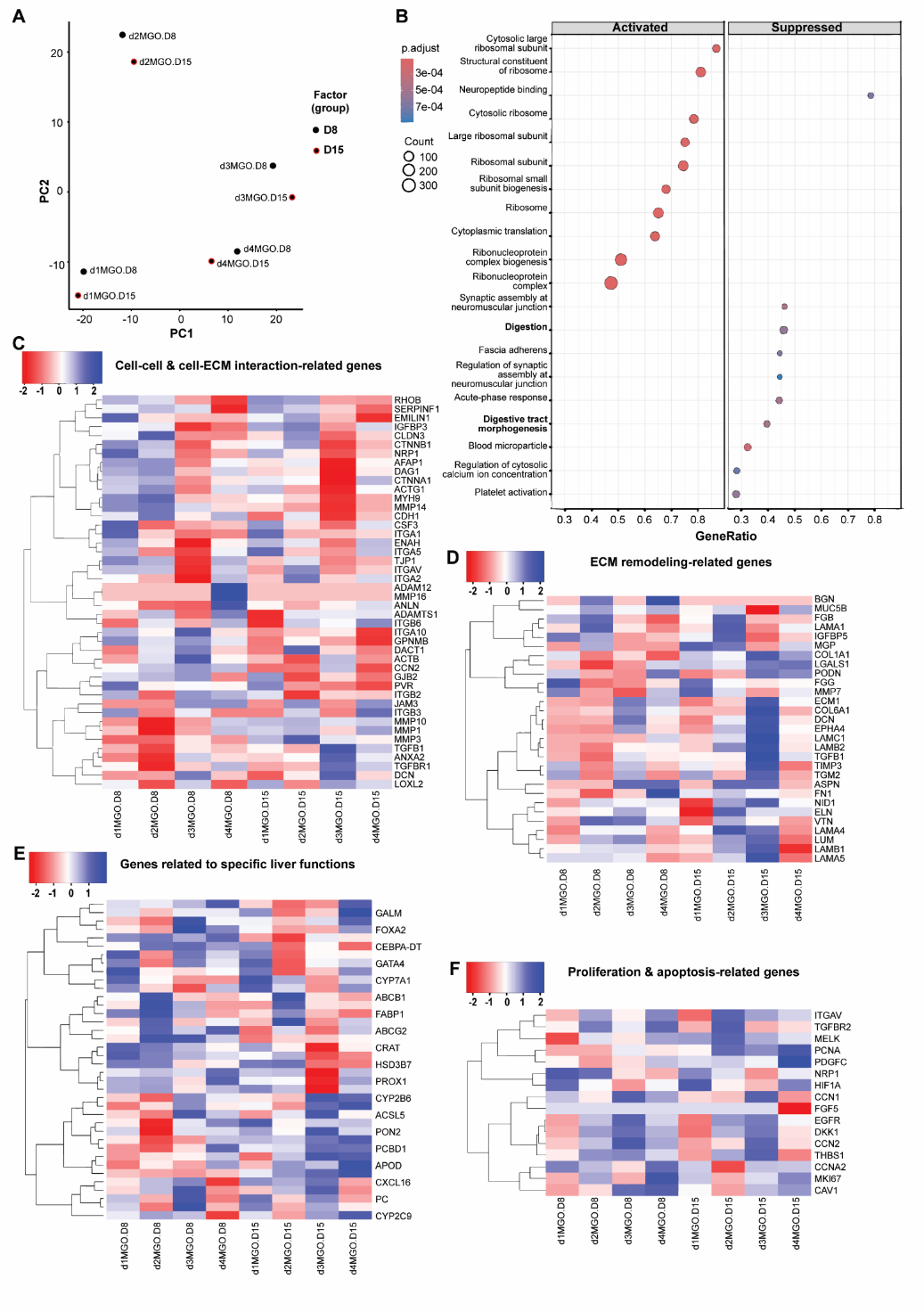


***Fig. S8. Transcriptomic comparison of organoid-only (O) condition between D8 and D15.*** *(A) PCA graph shows the contributions of the top two principal components to the variance in mRNA expression of BLTs on day 8 (D8) and day 15 (D15) of differentiation. Four donors (d1, d2, d3, d4) were applied. (B) GO analysis reveals the top involved signaling pathways for all genes comparing O samples on D8 and D15. Heatmaps were made as described above with previously reported genes that are associated with cell-cell & cell-ECM interaction (C), ECM-remodeling (D), specific liver functions (E), and proliferation & apoptosis (F). n=4*


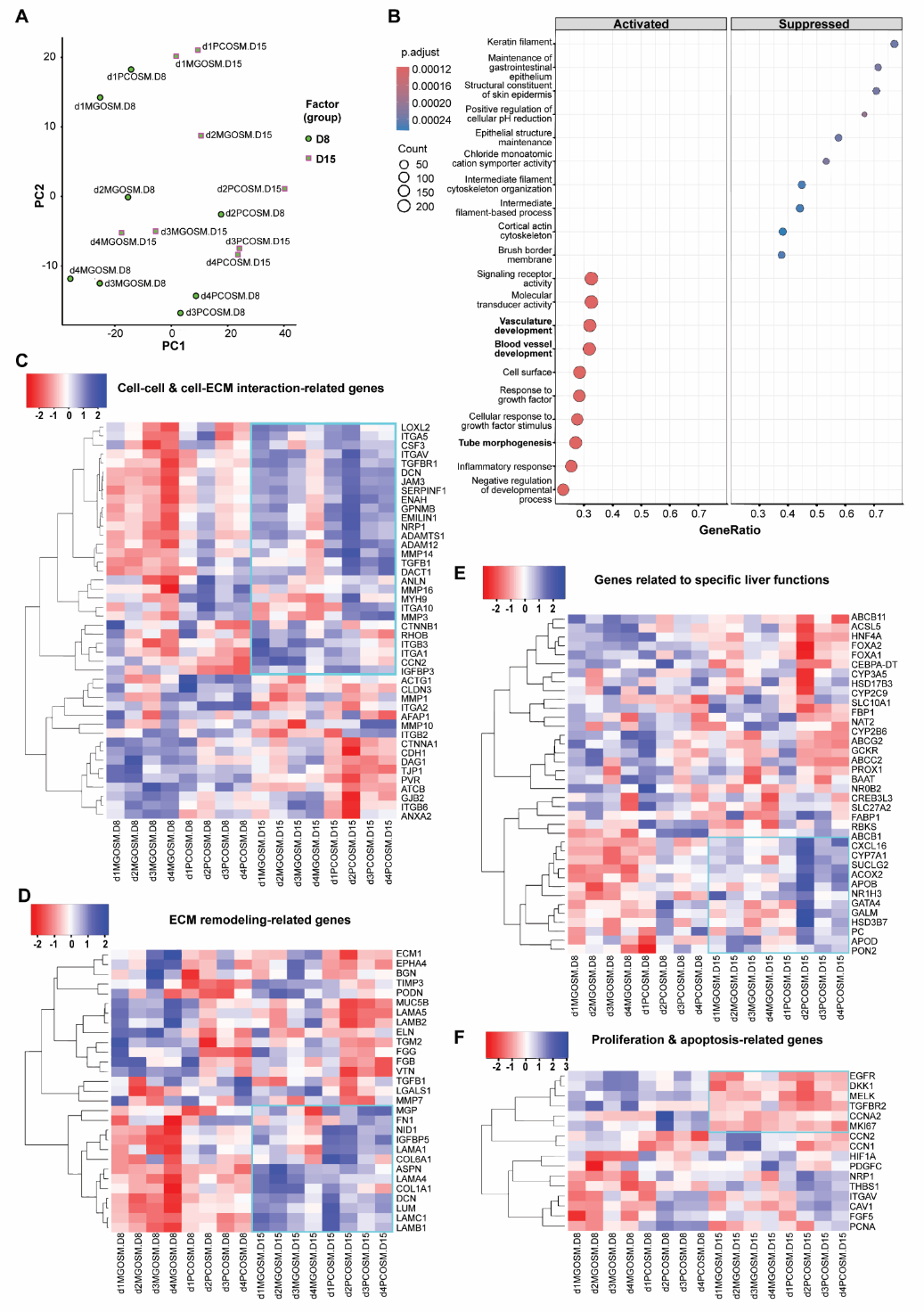


***Fig. S9. Transcriptomic comparison of the co-culture (OSM) condition between D8 and D15.*** *(A) PCA graph shows the contributions of the top two principal components to the variance in mRNA expression of OSM-derived BLTs on day 8 (D8) and day 15 (D15) of differentiation. Involved factors include two hydrogels, Matrigel (MG) and PIC + Collagen (PC), and four donors (d1, d2, d3, d4). (B) GO analysis reveals the top involved signaling pathways for all genes comparing OSM samples on D8 and D15. Heatmaps were made as described above with previously reported genes associated with cell-cell & cell-ECM interaction (C), ECM-remodeling (D), specific liver functions (E), and proliferation & apoptosis (F).* *n=4*


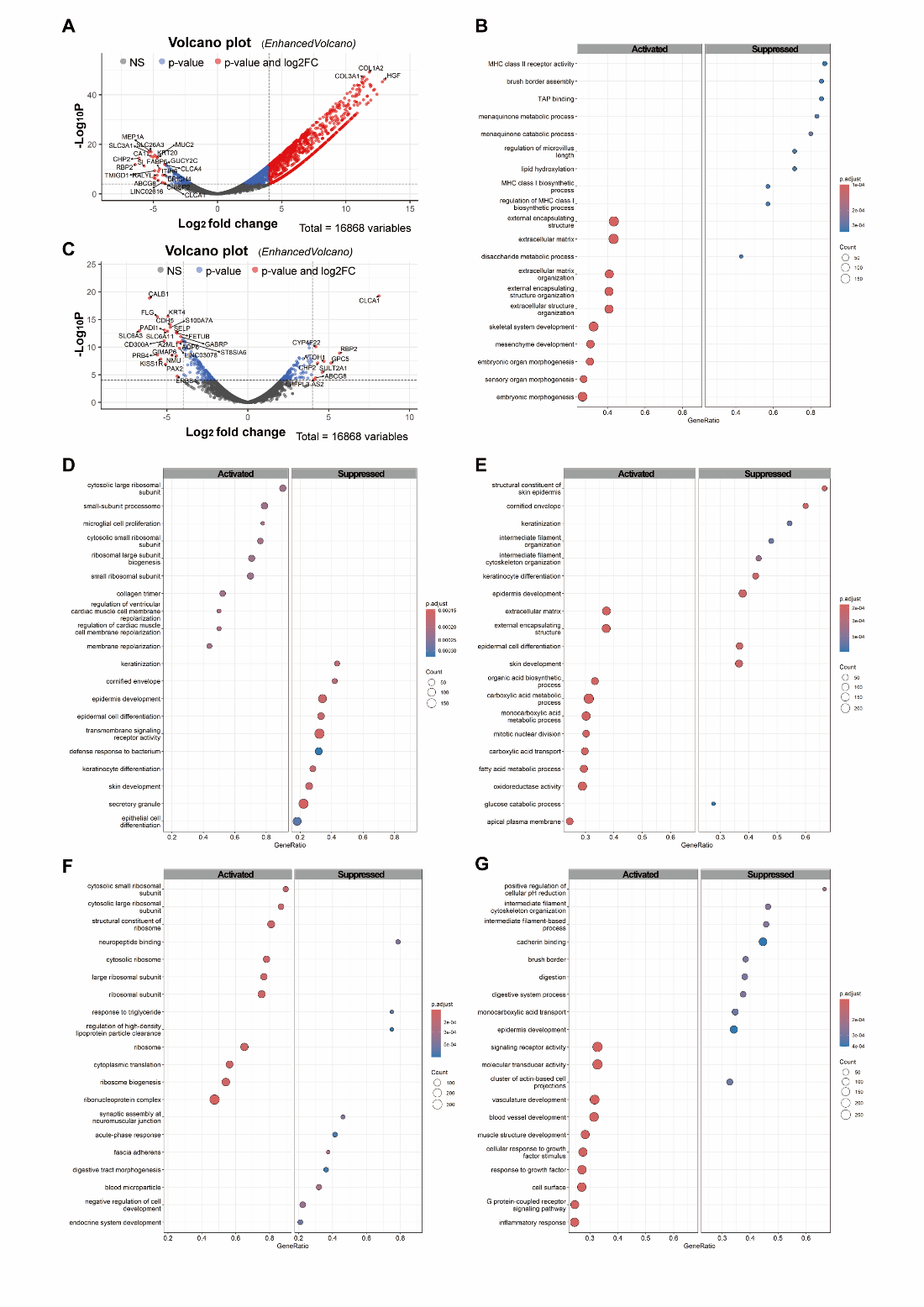


***Fig. S10. Donor-paired analysis of RNA-seq results.*** *(A-B) Donor-paired analysis related to Fig. 6B & S5A. (C-D) Donor-paired analysis related to Fig. 7B & S6A. (E-G) Donor-paired analysis related to Fig. S7B, S8B & S9B, respectively. n=4*

**Table S1. Selected candidate gene lists for heatmap.**

| **Gene name** | **ENSG ID** | **Gene name** | **ENSG ID** | **Gene name** | **ENSG ID** |
| --- | --- | --- | --- | --- | --- |
| **ECM remodeling-related genes** | | **Cell-cell & cell-ECM interaction-related genes** | | **Genes related to specific liver functions** | |
| *LGALS1* | ENSG00000100097 | *CLDN3* | ENSG00000165215 | TFs | |
| *LUM* | ENSG00000139329 | *LFA-1* | ENST00000477516 | *CEBPA* | ENSG00000245848 |
| *MMP7* | ENSG00000137673 | *VLA-5* | ENSG00000161638 | *CREB3L3* | ENSG00000060566 |
| *FN1* | ENSG00000115414 | *PVR* | ENSG00000073008 | *FOXA1* | ENSG00000129514 |
| *VTN* | ENSG00000109072 | *ITGB6* | ENSG00000115221 | *FOXA2* | ENSG00000125798 |
| *EPHA4* | ENSG00000116106 | *IGFBP3* | ENSG00000146674 | *GATA4* | ENSG00000285109 |
| *MGP* | ENSG00000111341 | *CDH1* | ENSG00000039068 | *HNF4A* | ENSG00000101076 |
| *DCN* | ENSG00000011465 | *ITGA1* | ENSG00000213949 | *NR1H3* | ENSG00000025434 |
| *IGFBP5* | ENSG00000115461 | *ITGA5* | ENSG00000161638 | *NR1I3* | ENSG00000143257 |
| *TGFB1* | ENSG00000105329 | *ITGB2* | ENSG00000160255 | *PCBD1* | ENSG00000166228 |
| *COL1A1* | ENSG00000108821 | *MMP1* | ENSG00000196611 | *PROX1* | ENSG00000117707 |
| *COL6A1* | ENSG00000142156 | *TGFBR1* | ENSG00000106799 | Glucose | |
| *ECM1* | ENSG00000143369 | *MMP3* | ENSG00000149968 | *GALM* | ENSG00000143891 |
| *LAMA1* | ENSG00000101680 | *MMP10* | ENSG00000166670 | *FBP1* | ENSG00000165140 |
| *LAMA4* | ENSG00000112769 | *CCN2* | ENSG00000118523 | *PC* | ENSG00000173599 |
| *LAMA5* | ENSG00000130702 | *ITGB3* | ENSG00000259207 | *RBKS* | ENSG00000171174 |
| *LAMB1* | ENSG00000091136 | *MMP16* | ENSG00000156103 | *SUCLG2* | ENSG00000172340 |
| *LAMB2* | ENSG00000172037 | *ADAMTS1* | ENSG00000154734 | Fat | |
| *LAMC1* | ENSG00000135862 | *DACT1* | ENSG00000165617 | *ACOX2* | ENSG00000168306 |
| *ELN* | ENSG00000049540 | *NRP1* | ENSG00000099250 | *ACSL5* | ENSG00000197142 |
| *FGB* | ENSG00000171564 | *DAG1* | ENSG00000173402 | *FABP1* | ENSG00000163586 |
| *NID1* | ENSG00000116962 | *MMP14* | ENSG00000157227 | *SLC27A2* | ENSG00000140284 |
| *FGG* | ENSG00000171557 | *EMILIN1* | ENSG00000138080 | *CRAT* | ENSG00000095321 |
| *ASPN* | ENSG00000106819 | *ITGAV* | ENSG00000138448 | Drug | |
| *PODN* | ENSG00000174348 | *JAM3* | ENSG00000166086 | *HSD17B3* | ENSG00000130948 |
| *PRG2* | ENSG00000186652 | *ITGA2* | ENSG00000164171 | *CYP2B6* | ENSG00000197408 |
| *TGM2* | ENSG00000198959 | *ADAM12* | ENSG00000148848 | *CYP2C9* | ENSG00000138109 |
| *MUC5B* | ENSG00000117983 | *GPNMB* | ENSG00000136235 | *CYP3A5* | ENSG00000106258 |
| *BGN* | ENSG00000182492 | *MYH9* | ENSG00000100345 | *GCKR* | ENSG00000084734 |
| *TIMP3* | ENSG00000100234 | *CTNNA1* | ENSG00000044115 | *NAT2* | ENSG00000156006 |
|  |  | *LOXL2* | ENSG00000134013 | Cholesterol | |
|  |  | *DCN* | ENSG00000011465 | *PON2* | ENSG00000105854 |
| **Autocrine & paracrine effect-related genes** | | *ANXA2* | ENSG00000182718 | *APOB* | ENSG00000084674 |
|  |  | *SERPINF1* | ENSG00000282307 | *APOD* | ENSG00000189058 |
| *FGF18* | ENSG00000156427 | *ACTG1* | ENSG00000184009 | *CXCL16* | ENSG00000161921 |
| *WNT16* | ENSG00000002745 | *TGFB1* | ENSG00000140682 | Bile synthesis | |
| *BMP2* | ENSG00000125845 | *RHOB* | ENSG00000143878 | *NR0B2* | ENSG00000131910 |
| *TGFB3* | ENSG00000119699 | *ACTB* | ENSG00000075624 | *ACOX2* | ENSG00000168306 |
| *BMP6* | ENSG00000153162 | *TJP1* | ENSG00000104067 | *CYP7A1* | ENSG00000167910 |
| *ACKR4* | ENSG00000129048 | *ANLN* | ENSG00000011426 | *BAAT* | ENSG00000276559 |
| *PGF* | ENSG00000119630 | *AFAP1* | ENSG00000196526 | *HSD3B7* | ENSG00000099377 |
| *CXCL8* | ENSG00000169429 | *DACT1* | ENSG00000165617 | Bile transport | |
| *CCL2* | ENSG00000108691 | *GJB2* | ENSG00000165474 | *ABCB11* | ENSG00000073734 |
| *ACKR3* | ENSG00000144476 | *CSF3* | ENSG00000108342 | *ABCB1* | ENSG00000085563 |
| *WNT2* | ENSG00000105989 | *ITGA10* | ENSG00000143127 | *ABCC2* | ENSG00000023839 |
| *IGFBP4* | ENSG00000141753 | *CTNNB1* | ENSG00000168036 | *ABCG2* | ENSG00000118777 |
| *WLS* | ENSG00000116729 | *CTNNA1* | ENSG00000044115 | *SLC10A1* | ENSG00000100652 |
| *IGFBP5* | ENSG00000115461 | *ITGA2* | ENSG00000164171 |  |  |
| *LTBP4* | ENSG00000090006 | *ENAH* | ENSG00000154380 |  |  |
| *NBL1* | ENSG00000158747 | **Proliferation & apoptosis-related genes** | | **Mechanotransduction-related genes** | |
| *WNT9A* | ENSG00000143816 |  |  |  |  |
| *CXCL2* | ENSG00000081041 | *Ki67* | ENSG00000148773 | *YAP1* | ENSG00000137693 |
| *TGFB2* | ENSG00000092969 | *CCNA2* | ENSG00000145386 | *PIEZO2* | ENSG00000154864 |
| *SOCS2* | ENSG00000120833 | *FGF5* | ENSG00000138675 | *PIK3CA* | ENSG00000121879 |
| *SOCS3* | ENSG00000184557 | *DKK1* | ENSG00000107984 | *NOTCH1* | ENSG00000148400 |
| *SOX9* | ENSG00000125398 | *EGFR* | ENSG00000146648 | *MRTFB* | ENSG00000186260 |
| *CXCL6* | ENSG00000124875 | *TGFBR2* | ENSG00000163513 | *MAPK8* | ENSG00000107643 |
| *SOX4* | ENSG00000124766 | *PDGFC* | ENSG00000145431 | *AKT1* | ENSG00000142208 |
| *FGF7* | ENSG00000140285 | *MELK* | ENSG00000165304 | *DDR2* | ENSG00000162733 |
| *CXCL3* | ENSG00000163734 | *NGF* | ENSG00000134259 | *ASMA* | ENSG00000143632 |
| *CRLF1* | ENSG00000006016 | *CAV1* | ENSG00000105974 | *HNF4A* | ENSG00000101076 |
| *CLCF1* | ENSG00000175505 | *HIF1A* | ENSG00000100644 | *CTNNB1* | ENSG00000168036 |
| *LIF* | ENSG00000128342 | *PCNA* | ENSG00000132646 | *ITGB1* | ENSG00000150093 |
| *FGFR3* | ENSG00000068078 | *CCN1* | ENSG00000142871 | *RHOA* | ENSG00000067560 |
| *CXCL1* | ENSG00000163739 | *THBS1* | ENSG00000137801 | *ROCK1* | ENSG00000067900 |
|  |  | *CCN2* | ENSG00000118523 | *CDH1* | ENSG00000039068 |
|  |  | *ITGAV* | ENSG00000138448 | *ITGB2* | ENSG00000160255 |
|  |  | *NRP1* | ENSG00000099250 | *ICAM-1* | ENSG00000090339 |
